# Genome Landscape and Evolutionary Plasticity of Chromosomes in Malaria Mosquitoes

**DOI:** 10.1371/journal.pone.0010592

**Published:** 2010-05-12

**Authors:** Ai Xia, Maria V. Sharakhova, Scotland C. Leman, Zhijian Tu, Jeffrey A. Bailey, Christopher D. Smith, Igor V. Sharakhov

**Affiliations:** 1 Department of Entomology, Virginia Tech, Blacksburg, Virginia, United States of America; 2 Department of Statistics, Virginia Tech, Blacksburg, Virginia, United States of America; 3 Department of Biochemistry, Virginia Tech, Blacksburg, Virginia, United States of America; 4 Program in Bioinformatics and Integrative Biology and Division of Transfusion Medicine, Department of Medicine, University of Massachusetts Medical School, Worcester, Massachusetts, United States of America; 5 Department of Biology, San Francisco State University, San Francisco, California, United States of America; 6 Drosophila Heterochromatin Genome Project, Lawrence Berkeley National Lab, Berkeley, California, United States of America; Texas A&M University, United States of America

## Abstract

**Background:**

Nonrandom distribution of rearrangements is a common feature of eukaryotic chromosomes that is not well understood in terms of genome organization and evolution. In the major African malaria vector *Anopheles gambiae*, polymorphic inversions are highly nonuniformly distributed among five chromosomal arms and are associated with epidemiologically important adaptations. However, it is not clear whether the genomic content of the chromosomal arms is associated with inversion polymorphism and fixation rates.

**Methodology/Principal Findings:**

To better understand the evolutionary dynamics of chromosomal inversions, we created a physical map for an Asian malaria mosquito, *Anopheles stephensi*, and compared it with the genome of *An. gambiae*. We also developed and deployed novel Bayesian statistical models to analyze genome landscapes in individual chromosomal arms *An. gambiae*. Here, we demonstrate that, despite the paucity of inversion polymorphisms on the X chromosome, this chromosome has the fastest rate of inversion fixation and the highest density of transposable elements, simple DNA repeats, and GC content. The highly polymorphic and rapidly evolving autosomal 2R arm had overrepresentation of genes involved in cellular response to stress supporting the role of natural selection in maintaining adaptive polymorphic inversions. In addition, the 2R arm had the highest density of regions involved in segmental duplications that clustered in the breakpoint-rich zone of the arm. In contrast, the slower evolving 2L, 3R, and 3L, arms were enriched with matrix-attachment regions that potentially contribute to chromosome stability in the cell nucleus.

**Conclusions/Significance:**

These results highlight fundamental differences in evolutionary dynamics of the sex chromosome and autosomes and revealed the strong association between characteristics of the genome landscape and rates of chromosomal evolution. We conclude that a unique combination of various classes of genes and repetitive DNA in each arm, rather than a single type of repetitive element, is likely responsible for arm-specific rates of rearrangements.

## Introduction

A growing number of studies demonstrate that chromosomal inversions facilitate genetic differentiation during speciation [1], [2]. An intriguing observation is that the rates of genome rearrangements in many organisms are chromosome sensitive [3], [4]. This fact suggests that certain chromosomes have an increased role in adaptation and evolution of species, including insect pests and disease vectors. Among insects, extensive studies of chromosomal evolution have been performed only on *Drosophila* [5], [6], [7], [8]. Although these studies provided important insights into the rates, patterns, and mechanisms of rearrangements, the evolutionary forces that govern the unequal distribution of rearrangements among chromosomes remain poorly understood. Malaria mosquitoes are an excellent system for studying the dynamics of chromosomal evolution because inversions are highly nonuniformly distributed among five chromosomal arms. In species of the *Anopheles gambiae* complex, 18 of the 31 common polymorphic inversions, associated with ecological adaptations, have been found on arm 2R suggesting the role of positive selection in accumulating inversions on the 2R arm. Only two polymorphic inversions have been found on the X chromosome within the *An. gambiae* complex [9]. A study of the distribution of 82 rare, mostly neutral, polymorphic inversions in *An. gambiae s.s.* found no inversions on the X chromosome, 67 inversions on the 2R arm, and only 15 inversions on the 2L, 3R, and 3L arms together [10]. Clustering of chromosomal polymorphism and cytological colocalization of multiple breakpoints on the 2R arm indicates that this arm is especially prone to rearrangements [9], [10]. In contrast to the polymorphic inversions, the majority of fixed inversions (5 of 10) were found on the X chromosome in the *An. gambiae* complex suggesting a role of these inversions in speciation. Although, the high density of fixed inversions on the sex chromosome was found within several mosquito species complexes [11], it is unclear whether the X chromosome rearranges rapidly on a larger evolutionary scale and whether it is enriched in genes important for speciation. Previous studies of chromosomal evolution using physical maps of distant *Anopheles* species, *An. albimanus*, *An. gambiae*, and *An. funestus* have demonstrated that paracentric inversions and whole-arm translocations are the major types of rearrangements and that the 2R arm has the fastest rate of inversion fixation among autosomes [12], [13]. However, low densities of markers on the physical maps of the X chromosomes in these studies preclude us from drawing a definite conclusion about the relative rate of sex chromosome evolution.

The high rate of rearrangements on the 2R arm could be explained by 2R-biased distribution of repetitive DNA capable of generating inversions. However, the transposable element (TE) density in the *An. gambiae* genome was found to be lowest on the 2R arm [14]; thus, it is not clear whether the molecular content could be associated with inversion polymorphism and fixation rates. Moreover, simple measuring of the TE densities is not a robust way for discerning differences between arms. Statistically sound comparisons of molecular features among chromosomal arms can be performed using Bayesian statistical models and procedures. Also, a study of other potentially rearrangement-causing elements, such as simple repeats and segmental duplications (SDs), is yet to be performed in *Anopheles*. Nucleotide base composition can also play a role in genome instability. For example, GC-rich regions have been implicated in forming fragile hotspot regions for rearrangements [15], [16]. In addition, the nonrandom pattern of genome rearrangements can be governed by the nuclear architecture. Because of the nonrandom nuclear organization, certain loci may colocalize and have increased opportunities to interact and generate specific rearrangements in certain types of tumors in humans [17], [18]. Additionally, other interactions may be inhibitory. Matrix-associated regions (MARs) of DNA can bind directly to lamin—a major protein of the nuclear envelope—and can potentially increase chromosome stability in the cell nucleus [19], [20].

*An. gambiae* and *An. funestus* are the major malaria vectors in Africa, and *An. stephensi* is the principal malaria vector in Asia. Taxonomically, these species belong to different series within the subgenus *Cellia*: *Pyretophorus* (*An. gambiae*), *Myzomyia* (*An. funestus*), and *Neocellia* (*An. stephensi*) [21]. A comparative study of mitochondrial genomes suggested that *An. gambiae* and *An. funestus* diverged from each other at least 36 million years ago [22]. Interestingly, the common polymorphic inversions tend to cluster on the chromosomal arm 2R in all three species [9], [23], [24], [25], suggesting that natural selection has a better chance to operate on the genetic content of this arm. The common inversions 2Rb, 2Rbc, 2Rcu, 2Ru, 2Rd, and 2La of *An. gambiae* are frequent in the arid Sahel Savanna and almost absent in humid equatorial Africa [9]. It has been argued that these inversions confer adaptive fitness to the drier environment [10], [26]. Therefore, it would be interesting to see if the 2R and 2L arms are enriched in genes that could be responsible for this adaptation. A comparison of sizes between rare and common polymorphic inversions has revealed that common inversions are less frequent at shorter lengths [10], [27], reflecting a smaller selective advantage when an inversion captures fewer genes [28]. This model predicts the positive correlation between gene density and the abundance of common inversions in a chromosomal arm.

Here, we developed a physical map for an Asian malaria mosquito, *Anopheles stephensi*, and compared gene orders among *An. gambiae*, *An. funestus*, and *An. stephensi*. We present the results of the Bayesian analysis of the genome landscapes and their association with the nonrandom distribution of chromosomal rearrangements in malaria mosquitoes. Our study revealed that the sex chromosome and autosomes have different patterns of relationships between inversion fixation and polymorphism. We also demonstrated that the rapidly and slowly evolving chromosomal arms have very distinct genome landscapes characterized by distinctly enriched gene subpopulations and classes of repetitive DNA.

## Results

### A 1-Mb-resolution physical map for *An. stephensi*

Availability of the genome sequence for *An. gambiae* [14] and physical maps for *An. funestus* [12], [29] and *An. stephensi* (this work) enabled a fresh perspective on the relationships between the genome landscape and evolutionary rates. In this study, we mapped 231 DNA markers to the *An. stephensi* chromosomes at a density of 1 marker/megabase (Mb) based on the mapped *An. gambiae* genome assembly [14], [30]. Table S1 shows chromosomal positions of the DNA clones mapped in this study, as well as in previous studies [12], [14], [29], [31], [32]. We performed a test on the uniformity of marker distribution in *An. gambiae*, *An. stephensi*, and *An. funestus* using the *Χ* ^2^ statistic. The distribution of markers was shown to be uniform for each arm and each species (Table S2). Comparative mapping established arm homologies among the three species; found no evidence for inter-arm transposition events, pericentric inversions, or partial–arm translocations (Table S1); and confirmed that whole-arm translocations and paracentric inversions are common rearrangements among species in the subgenus *Cellia* [12], [21].

### Pattern and rates of inversion fixation in the subgenus *Cellia*

**Figure 1:**
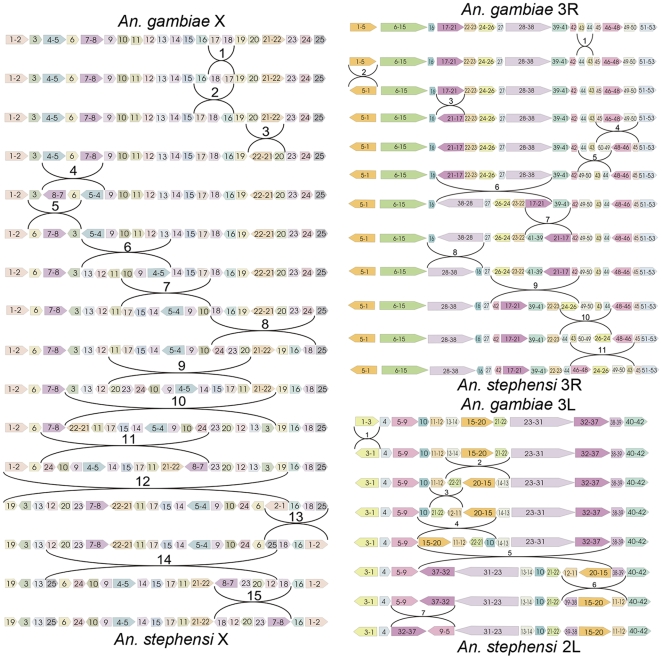
The GRIMM scenario of gene order transformation between *An. gambiae* and *A. stephensi*. Relative position and orientation of the conserved syntenic blocks (CSBs) are shown by colored blocks. Numbers within the blocks indicate markers physically mapped to polytene chromosomes. Numbers over brackets show inversion steps. The telomere ends are on the left.

We calculated the minimum number of inversions between *An. gambiae* and *An. stephensi* using the order of mapped markers (Table S1) and the Genome Rearrangements In Man and Mouse (GRIMM) program without assuming directionality of the markers [33]. GRIMM software uses the Hannenhalli and Pevzner algorithms for computing the minimum number of rearrangement events and for finding optimal scenarios for transforming one genome into another. A minimum of 15 rearrangement events are needed to transform the 24.4-Mb-long X chromosome of one species into the other. In contrast, only 11 and 7 inversions are required to transform the 53.2-Mb-long 3R arm and the 42-Mb-long 3L arm, respectively (Figure 1). The 2R and 2L arms had 29 and 16 fixed inversions, respectively (Figure S1, S2). When normalized to account for differences in chromosome length, the X chromosome had the highest density of fixed inversions of any chromosome (Figure S1, S2, Table 1). The highest level of inversion fixation on the X chromosome was also found for the analogous comparison of *An. gambiae* and *An. funestus* (Table S3). We calculated number of breaks per Mb under the assumption that there is no breakpoint re-use and no inversions at the very ends of chromosomes (Table 1, Table S3). The rearrangement scenarios provided by the GRIMM program had breakpoint reuses and yielded lower number of breaks per Mb (Figure 1, S1, S2). However, the actual breakpoint reuse cannot be identified at 1Mb density of markers physically mapped to chromosomes.

**Table 1:** Inversion fixation rates between *An. stephensi* and *An. gambaie* calculated from GRIMM analysis of gene order.

Chromosome arm	The number of inversions, *n*	The length of chromosomal arm, *G* (Mb)	The number of inversions per 1 Mb	The number of breaks per 1 Mb
X	15	24.393	0.615	1.230
2R	29	61.545	0.471	0.942
2L	16	49.364	0.324	0.648
3R	11	53.201	0.207	0.414
3L	7	41.963	0.167	0.334

As another approach to inversion frequency, we also employed an analysis of conserved syntenic blocks (CSBs), which are defined as the regions with the same order and distance between at least two markers (Table S1). In order to provide better estimates of CSBs, we further developed the Nadeau and Taylor method [34]. Using the adapted Bayesian Nadeau and Taylor analysis, we found the posterior mean, standard error, 95% credible interval, and Maximum A Posteriori (MAP) estimate for the mean length of CSBs (See Methods). These lengths (X, 0.600 Mb; 2R, 1.315 Mb; 2L, 1.712 Mb; 3R, 3.756 Mb; and 3L, 2.412 Mb) (Table S4) were also used to infer the number of fixed inversions between *An. gambiae* and *An. stephensi*. If each inversion requires two disruption events, then *n* inversions result in *2n+1* conserved segments. The number of CSBs was calculated by dividing the total length of the arm by the mean length of the CSB (Table 2). Nadeau and Taylor analysis was not applied to *An. gambiae* and *An. funestus* because no CSBs were detected on the X chromosome. However, the GRIMM analysis inferred the level of rearrangement between *An. gambiae* and *An. funestus* (Table S3). Given that *An. gambiae* and *An. funestus* diverged from each other at least 36 million years ago [22], the rate of genome rearrangement in the subgenus *Cellia* for 1 Mb mapping density is 0.006–0.01 disruptions per 1 Mb per million years per lineage.

**Table 2:** Inversion fixation rates between *An. stephensi* and *An. gambiae* calculated from the Nadeau-Taylor analysis of the mean length of CSBs.

Chromosome arm	The mean length of CSBs, *L* (Mb)	The length of chromosomal arm, *G* (Mb)	The number of CSBs, *M = G/L*	The number of inversion, *n* = (*M*−1)/2	The number of inversions per 1 Mb	The number of breaks per 1 Mb
X	0.600	24.393	40.652	19.826	0.813	1.626
2R	1.315	61.545	46.791	22.895	0.372	0.744
2L	1.712	49.364	28.830	13.915	0.282	0.564
3R	3.756	53.201	14.165	6.583	0.124	0.247
3L	2.412	41.963	17.395	8.198	0.195	0.391

**Figure 2:**
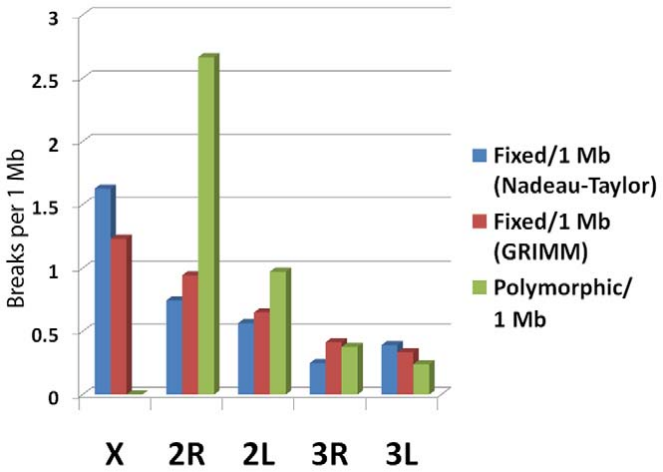
The contrasting patterns of the X chromosome and autosome evolution. The fastest evolution of the X chromosome and parallelism between the extent of inversion polymorphism and inversion fixation rates on the autosomes are shown. The number of breakpoints of fixed inversions is calculated per 1 Mb from Nadeau-Taylor analysis (the blue bar) and GRIMM analysis (the red bar). The number of breakpoints of all polymorphic inversions in *An. gambiae* and *An. stephensi* is combined and calculated per 1 Mb (the green bar).

Both Nadeau-Taylor and GRIMM analyses revealed that the X chromosome had the highest rate of inversion fixation and that the 2R arm evolved faster than other autosomes. The fastest evolution was in the X chromosome, which was in conflict with the absence of polymorphic inversions on the X chromosome in all three species [10], [29], [32]. In contrast, inversion fixation rates on autosomes were well correlated with the distribution of polymorphic inversions in *An. gambiae*—*An. stephensi* (correlation coefficients were 0.98 and 0.89 for GRIMM and Nadeau-Taylor analyses, respectively), when all polymorphic inversions in *An. gambiae* [10] and *An. stephensi* [23], [35], [36] were combined (Figure 2). The correlation coefficient between fixed and polymorphic inversions in *An. gambiae*—*An. funestus* [12], [29] was 0.87 (Figure S3).

### Distribution of repetitive elements and genes in chromosomes of *An. gambiae*

**Figure 3:**
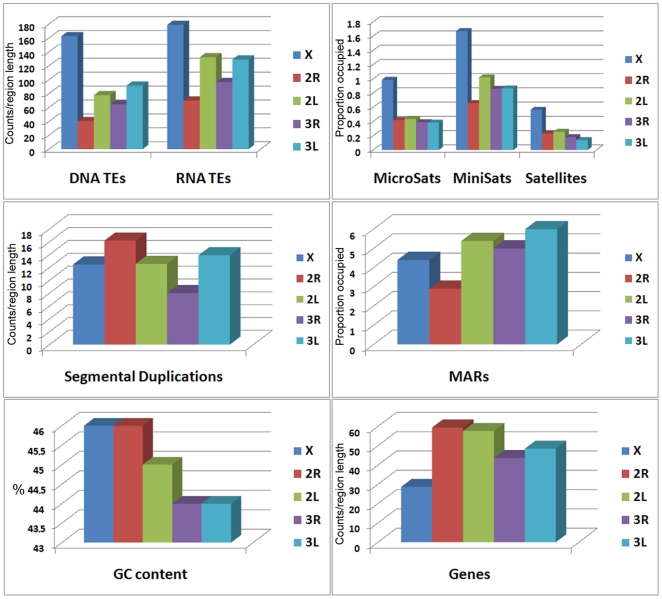
Median values of density and coverage of molecular features in chromosomes of *An. gambiae*. Counts per 1 Mb are given for DNA TEs, RNA TEs, regions involved in SDs, and genes. Percentage of region length occupied per 1 Mb are indicated for microsatellites, minisatellites, satellites, and MARs.

We applied a Bayesian statistical model and procedure for discerning differences between arms in molecular features, such as DNA-mediated TEs (DNA TEs), RNA-mediated TEs (RNA TEs), SDs, micro- and minisatellites, satellites, MARs, and genes. For this analysis, we incorporated data that distinguishes both the counts and the overall basepair coverage for each molecular feature in the genomic windows of each of the five chromosome arms. Dominant model selection procedures gave us the ability to compare all possible competing models and to select between parsimonious models by maximizing the posterior distribution. For DNA TEs, RNA TEs, microsatellites, minisatellites, satellites, and genes, we found that each of the arms showed significant differences (Figure 3, Table S5). For MARs, we found that the model with arms 2L = 3L and the model with 2L = 3R = 3L are almost equally possible. For the regions involved in SDs, we found little support for the difference between the model with X = 2L and the model with all arms being different. In all cases, the 2R arm showed clear differences and did not show patterns that match any of the other arms.

The X chromosome had the highest density of TEs and the highest coverage of microsatellites, minisatellites, and satellites. The 2R arm had the highest density of genes and regions involved in SDs but had the lowest densities of TEs and the lowest coverage of minisatellites and MARs (Figure 3). In contrast to all other repeats, MARs were concentrated in arms 2L, 3R, and 3L. We found a negative correlation between the rates of fixed inversions from GRIMM analysis and MARs coverage (r = −0.766), suggesting a role for nuclear architecture in controlling the rearrangements. The coefficients of correlation between inversion fixation rates and the densities or coverage of other individual molecular elements were the following: 0.274 for DNA TEs, 0.266 for RNA TEs, −0.193 for SDs, 0.824 for microsatellites, 0.562 for minisatellites, and 0.812 for satellites. If we assume that all these repetitive elements except MARs have an equal positive impact on chromosomal breakage, then we can consider mean ranks of their density/coverage as a function of inversion fixation rate. The average mean ranks for all repeats without MARs were 3.914, 2.575, 2.989, 2.663, and 2.860 for X, 2R, 2L, 3R, and 3L, respectively (Table S5). The coefficient of correlation between inversion fixation rates and the average mean ranks was only 0.662. Also, we assumed that MARs have a negative impact on chromosomal breakage, and we considered mean ranks of MAR coverage as a function of genome stability. Therefore, to obtain a resulting effect of all repetitive elements on inversion fixation rates, we subtracted the mean ranks for MARs from the average mean ranks for all other repeats and obtained 1.213, 0.231, −0.337, −0.391, and −0.714 for X, 2R, 2L, 3R, and 3L, respectively. The recalculated correlation coefficient value between these mean ranks and the inversion fixation rates increased significantly up to 0.962. These results demonstrate a strong association between the observed inversion fixation pattern and the possible combined effect of MARs and other repeats on chromosome instability.

**Figure 4:**
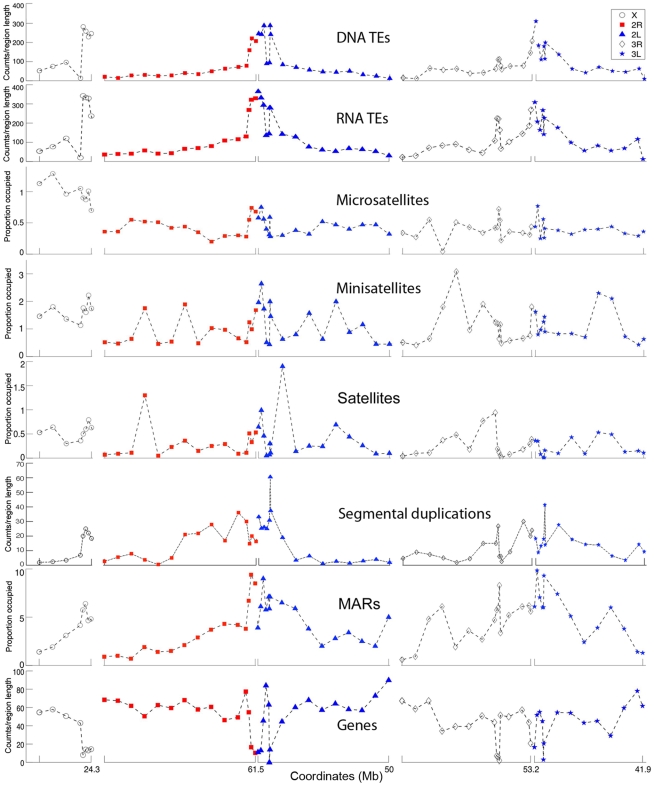
Genome landscapes of the *An. gambiae* chromosomal arms. Median counts per 1 Mb are given for DNA TEs, RNA TEs, regions involved in SDs, and genes. Percentage of region length occupied per 1 Mb is indicated for microsatellites, minisatellites, satellites, and MARs. Median values of density and coverage of molecular features are displayed as 5 Mb intervals in euchromatin and <1 Mb intervals in heterochromatin. The coordinates and orientation of each arm are the following: X: 0 Mb—telomere, 24.3 Mb—centromere; 2R: 0 Mb—telomere, 61.5 Mb—centromere; 2L: 0—centromere, 50 Mb—telomere; 3R: 0 Mb—telomere, 53.2 Mb—centromere; 3L: 0 Mb—centromere, 41.9 Mb—telomere.

In addition to the arm differences, we analyzed the distribution of molecular features within chromosomal arms. There was a uniformly low concentration of TEs in euchromatin with peaks being in pericentric and intercalary heterochromatin. The distribution of gene densities had the opposite pattern. MARs were found concentrated in the pericentric regions of all arms, but they were also abundant in euchromatiic regions of the 2L, 3R, and 3L arms. We detected the highest density of regions with SDs in the proximal half of the 2R arm where the breakpoint-rich area is located [10] (Figure 4). The correlation coefficient between the densities of breakpoints and regions involved in SDs in 5-Mb intervals within 50 Mb of the euchromatic part of 2R was 0.9091, suggesting an arm specific involvement of SDs in inversion formation rather than a genome-wide impact.

### AT/GC content of the *An. gambiae* chromosomes

We analyzed empirical median AT content and found it equal to 0.46, 0.46, 0.55, 0.56, and 0.56 for the X, 2R, 2L, 3R, and 3L arms, respectively. To statistically compare AT/GC content among chromosomal arms, we quantified the level of uncertainty associated with these numbers and calculated probabilities that respective arms have a higher AT content than the X chromosome, which was used as the baseline reference for all comparisons. The probabilities were 0.677 (2R), 0.855 (2L), 0.871 (3R), and 0.888 (3L). These results demonstrate that 2L, 3R, and 3L have a moderate increase in AT content over the X chromosome; whereas, the 2R arm has only a mild increase. The correlation coefficient between inversion fixation rates and the GC content was 0.954.

### Gene ontology analysis

**Figure 5:**
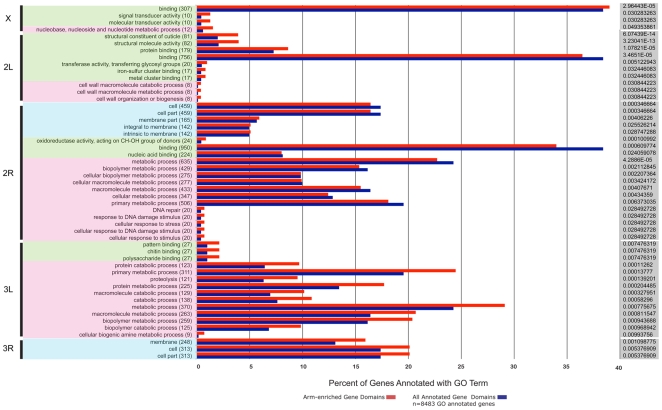
Overrepresented GO terms enriched on each chromosomal arm of the *An. gambiae* genome assembly. The percentages of arm-enriched (red) genes containing the listed GO biological process (pink shading), cellular location (blue shading), and molecular function (green shading) terms are compared to the percent of genes in the whole genome matching that term. Numbers in parentheses refer to the actual number of arm-enriched genes annotated with the listed GO domain. P-value significance scores, as determined by GO-Term-Finder, are shown to the right (grey shading).

We used Gene Ontology (GO) terms [37] to characterize gene content of individual chromosomal arms of *An. gambiae*. The frequencies of GO terms assigned to genes in chromosomal arms were compared to frequencies for all GO-annotated genes in the peptide dataset of *An. gambiae* (Figure 5). We found significant enrichment of GO terms in molecular function category on the X chromosome including molecular transducer activity (10 genes), signal transducer activity (10 genes), and binding (307 genes). Moreover, 12 genes on the X chromosomes were involved in nucleobase, nucleoside, and nucleotide metabolic processes representing a significant enrichment of the GO biological process. Chromosomal arm 2L had overrepresentation of several gene types including those encoding for proteins involved in structural constituent of cuticle, structural molecule activity, and protein binding (molecular function). In addition, 2L was enriched in GO terms of biological process: cell wall macromolecule catabolic process, cell wall macromolecule metabolic process, and cell wall organization or biogenesis. Arm 2R had overrepresentation of the following GO terms: membrane part, transmembrane proteins, proteins intrinsic to the membrane (cellular location), oxidoreductase activity, acting on CH-OH group of donors (molecular function), DNA repair, cellular response to stimulus, cellular response to DNA damage stimulus, cellular response to stress, and response to DNA damage stimulus (biological process). Chromosomal arm 3L was enriched in GO terms related to binding (molecular function) and metabolic/catabolic processes (biological process). Finally, 3R had an overrepresentation of several gene types including those encoding for proteins located in the membrane, cell, and cell parts (cellular location).

## Discussion

Our study revealed contrasting patterns of sex chromosome and autosome evolution. We demonstrated that the sex chromosome has the highest rate of inversion fixation, which is in contrast with the absence of polymorphic inversions on the X chromosome in the studied species (Figure 2, S3). The paucity of polymorphic inversions on the X chromosome could be a consequence of a low rate of origin of inversions. However, the X chromosome had the highest densities of TEs, microsatellites, minisatellites, and satellites, which are known for their roles in the origin of inversions [38], [39], [40]. The excess of fixed inversions, as compared to a deficit of polymorphic inversions, on the X chromosome has been documented in other insect species [11], [41]. A classical work has shown that the fixation rate of underdominant and advantageous partially or fully recessive rearrangements should be higher for the X chromosome (due to the hemizygosity of males) than for the autosomes [41]. It is possible that strong sex-specific selection favors hemizygous males carrying the X inversion, which is underdominant in females. Ayala and Coluzzi proposed that genes responsible for reproductive isolation of mosquito species should be located on the X chromosome [1]. Indeed, the X chromosome has a disproportionately large effect on male and female hybrid sterility and inviability in *An. gambiae* and *An. arabiensis* [42], [43]. The rapid evolution of sterility and inviability genes captured by polymorphic inversions on the X chromosome may cause a selection against inversion heterozygotes. From a vector control point of view, if heterozygote inversions on the X chromosome have a deleterious effect on viability and reproduction of mosquitoes, then they could be introduced artificially into the vector population to reduce its size. Our study of GO term distribution suggests that the X chromosome is enriched in genes that may be involved in premating isolation, such as genes encoding for proteins with molecular and signal transduction activity. Signal transduction is a crucial component of olfaction that plays a major role in mate recognition. For example, X-linked genes encoding for signal transduction proteins were differentially expressed between virgin females of two incipient species of *An. gambiae* that differ in swarming behavior [44]. Rapid generation and fixation of inversions on the X chromosome may facilitate speciation in *Anopheles* by differentiating alleles inside of the inverted regions as has been shown in *Drosophila* [45].

Unlike the X chromosome in insects, the eutherian X chromosome had its gene order conserved during 105 million years of evolution, probably reflecting strong selective constraints posed by the X inactivation system in mammals [46]. A study of the opossum genome revealed that the evolution of the X chromosome inactivation was associated with suppression of large-scale rearrangements in eutherians [47]. Conversely, rapidly evolving sex chromosomes in insects have a dosage compensation system. Because the X chromosome in *Drosophila* males recruits fewer histones and possesses an “open” chromatin [48], it may be more sensitive to breakage [16] and, thus, more prone to rearrangements.

In contrast to the X chromosome, the 2R and 2L arms of *An. gambiae* and their homologous arms in *An. stephensi* and *An. funestus* harbor polymorphic inversions associated with ecological adaptations [9], [23], [24]. Natural selection has been implicated in fixation of the 2Rj inversion during ecotypic speciation in *An. gambiae* [49]. Adaptive alleles or allelic combinations can be maintained within a polymorphic inversion by suppressing recombination between the loci [2], [50]. It has been demonstrated that adaptive inversions are less frequent at shorter lengths [10], [27], reflecting a smaller selective advantage when an inversion captures fewer genes [28]. Therefore, we predicted that chromosomal arms rich in polymorphic inversions (2R, 2L) would have higher gene densities. This prediction was met; moreover, the polymorphic inversion-poor X chromosome had the lowest gene density (Figure 3, Table S5). Similarly, the polymorphic inversion-rich chromosomal elements C and E have higher gene densities than the rest of the genome in *Drosophila* [5]. These observations highlight the fundamental differences between the evolutionary dynamics of the sex chromosome and autosomes. The high rate of sex chromosome evolution is being achieved by the rapid generation and fixation of inversions without maintenance of a stable inversion polymorphism. In contrast, the high rate of the autosomal evolution results from the high level of inversion polymorphism maintained by selection acting on gene-rich chromosomal arms. The increase of gene density in rearrangement-rich regions of autosomes was also found in vertebrates [15], [51], [52] suggesting the general applicability of the principle “from polymorphism to fixation” to autosomal evolution.

The polymorphic inversions 2Rb, 2Rbc, 2Rcu, 2Ru, 2Rd, and 2La of *An. gambiae* are associated with adaptation of mosquitoes to the dry environment [9]. Cuticle seems to play a major role in desiccation resistance of embryo and adult mosquitoes [26], [53]. These observations suggest an exciting possibility that genes involved in the cuticle development may be disproportionally clustered on the 2R and 2L arms. Our study of GO terms provides evidence that 2L is indeed enriched with genes involved in the structural integrity of a cuticle while the 2R arm has overrepresentation of genes involved in cellular response to stress (e.g., temperature, humidity) and in building membrane parts (Figure 5). These data support the role of natural selection in maintaining polymorphic inversions associated with ecological adaptations.

If nonrandom origin of inversions can be attributed to unequal density of repetitive DNA among chromosome arms, we would predict higher densities of break-causing elements on faster evolving arms. Indeed, the X chromosome had the highest densities of DNA and RNA TEs (Figure 3), which can potentially generate inversions [38], [39]. In addition, the X chromosome had the highest microsatellite, minisatellite, and satellite DNA content. Simple repeats have been shown to play a role in the formation of hairpin and cruciform structures, which can cause double-strand DNA breaks and rearrangements [40]. In *Drosophila*, the fastest evolving X chromosome has the highest densities of microsatellites and TEs [5], [54]. Although, the role of TEs in the origin of individual inversions was demonstrated earlier [38], [39], [55], [56], [57], the more recent sequencing of breakpoints discovered alternative mechanisms of inversion generation [6], [7], [8], [58]. SDs have been implicated in inversion generation in mosquitoes and mammals [59], [60] and are considered as a marker of genome fragility [61]. Our study showed that the most rapidly evolving autosomal arm 2R had the lowest density of TEs but the highest density of regions with SDs (Figure 3). Importantly, the regions involved in SDs were clustered in the proximal half of the 2R arm (Figure 4) where the majority of inversion breakpoints are found [10]. We also demonstrated that the 2R arm has the lowest coverage of MARs, which can potentially mediate interactions of specific chromosome sites with the nuclear envelope [19], [20]. Three-dimensional organization of chromosomes in the nuclear space can affect rearrangement rates by facilitating or hindering interchromosomal interactions [17], [18]. In agreement with this statement, MARs were found accumulated in the slowly evolving 2L, 3R, and 3R arms (Figure 3). We propose that multiple attachments of 2L, 3R, and 3L to the nuclear envelope make rejoining different breaks and forming inversions more difficult despite the abundance of TEs and simple repeats in these arms (Figure S4). Finally, we demonstrated that the *An. gambiae* X chromosome and 2R arm have the highest G+C content. GC-rich regions have been implemented in forming hotspots for chromosome rearrangements [15], [16] because of their propensity to form Z-DNA, hairpin loops, and other unstable structures that are capable of generating double-strand breaks [62]. Interestingly, our GO term analysis demonstrated that the X chromosome is enriched with nucleobase, nucleoside, and nucleotide metabolic processes and that the 2R arm has overrepresented gene clusters involved in DNA damage repair. It is possible that these GO term enrichments have evolved in response to high rates of DNA breakage on the X and 2R chromosomes.

Our study has shown that because of the paucity of pericentric inversions and partial-arm translocations in mosquito evolution, the genome landscapes and evolutionary histories of individual arms are different. The results demonstrated a strong association between the genome landscape characteristics and the rates of chromosomal evolution. We conclude that a unique combination of various classes of genes and repetitive DNA in each arm, rather than a single type of repetitive element, is likely responsible for arm-specific rates of rearrangements. These findings call for a reevaluation of the genomic analyses, which must be performed on an arm-by-arm basis using sequences physically mapped to the chromosomes.

## Methods

### Mosquito strain and physical mapping

For the physical map development, we used the Indian wild-type strain of *An. stephensi*. Chromosomal preparations from ovaries of half-gravid females and fluorescent in situ hybridization experiments were performed as described previously [12]. *An. stephensi*, *An. gambiae*, and *An. funestus* cDNA and BAC clones were hybridized to polytene chromosomes of *An. stephensi* (Table S1). Localization of a signal was done using a standard cytogenetic map for *An. stephensi* [32]. The BLASTN and BLASTX algorithms were used to identify homologous sequences in the *An. gambiae* genome, which is available at VectorBase [63].

### Test of uniformity of marker distribution

In order to determine if the marker distribution, along each chromosome arm, is distributed uniformly, we considered the 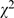 statistic:
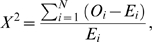
where *N* denotes a number of equally spaced bins. Under the null hypothesis (in this case, the distribution is uniform), 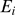 is the expected number of observations and 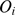 is the the observed number. Under large sample sizes, with each bin observed count having a sufficiently high count, 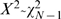. Large values of this statistic correspond to large deviations from the null. Analyses of distributional fit are often based on p-values, where the hypothesis is rejected when the p-value is under some predetermined threshold. However, these p-values (based on 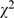 asymptotics) are only reliable under large sample sizes. Some of the chromosomes exhibit low marker counts (specifically the X chromosome), hence simulated p-values, based on bootstrap replications (100,000) are also provided. Under large sample sizes, bootstrap and asymptotic p-values will coincide.

Bin counts *N* were determined so that the each expected bin count was at least 5.

### Bayesian analysis of the Nadeau and Taylor model

We briefly review the method developed by Nadeau and Taylor (N-T) [34]. Letting *r* denote the range of observed marker lengths (as defined by the presence of two or more syntenic markers), N-T have shown the length of each marker to be
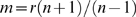
where 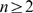 are the number of markers in each sytnteny region. We emphasize that *m* is the length of each region, given that it has been defined by at least two markers (as opposed to an unbiased length). N-T used a Poisson distribution for marker counts in order to account for this bias. Explicitly, the probability of observing at least two markers is
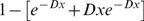
where *D* is the density (of all) markers in the genome, and *x* is the length of the conserved region. The density (*D*) is computed by: *D = T/G*, where *T* is the number of markers, and *G* is the genome length. Using this, N-T obtain the (un-normalized) sampling density for the length of each conserved block as
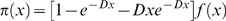
where 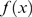 is the sampling density for the length (given that it is observed) of each region. N-T specify that 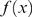 has an exponential distribution
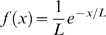
where *L* is the average length of each conserved segment. The analysis goal was to obtain an estimate of *L*. N-T have adopted a Method of Moments (MOM) approach for their estimation procedure. Under large sample sizes, it can be derived that
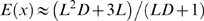
where 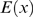 is obtained via the sample mean of the transformed lengths (given by equation 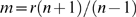). 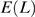 is obtained by back solving for *L*. 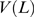 is obtained via the large sample estimate
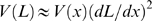
where 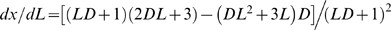. While the model adopted by N-T is useful for modeling the length of conserved chromosomal regions, the moments based estimation approach can lead to unreliable inferences.

Previously, we applied the N-T model to find the expected length of conserved synteny regions. After model fitting, we proceeded in diagnostically checking the model to see if it accurately represents our observed data trends. Through a *leave one out* cross validation procedure, under the described large sample approximations, a confidence region for the CDF (based on the fit parameters) was constructed. While the trend found in the data approximately matches that of the model, the expected 5% error rate was dramatically exceeded (29.6%). This excessive error rate could have occurred for either of two reasons. 1) The model is inappropriate for out data, or 2) the asymptotic approximations to the mean and variance are performing badly. In our case, we believe the variance estimates are simply underestimated. It should be noted that if we did have a larger data set, the problem incurred in (2) would diminish. In general, since sample sizes are fixed (for a given experiment), we will adopt a Bayesian inferential framework for overcoming the asymptotic deficiencies observed in the moments based approach. For notation, let us denote the model by
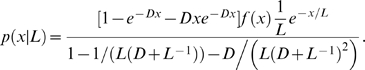
Formally, in a Bayesian analysis, one constructs a distribution on the parameter space *L*, given the data set 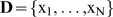. This distribution is referred to as a *posterior* distribution, and explicitly follows as
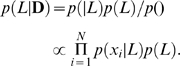
The distribution *p(L)* is called the *prior* distribution and is used to model beliefs about *L*, before observing the data. For our purposes, we used 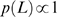, which represents (in this case) neutral beliefs about *L*, and doesn't favor any particular values *L*. While the choice of prior is quite flexible, the choice presented here makes the posterior have the same form as the *likelihood*. From this, we will obtain a full distribution for *L*, which will not rely on asymptotic approximations (The original framework simply provides an estimated mean and variance, which are valid under large sample sizes). Through Markov chain Monte Carlo, we obtain the posterior distribution for *L*. From this, we find the posterior mean, standard error, 95% credible interval, and Maximum A Posteriori (MAP) estimate for *L*, which are tabulated in Table S4. We assess the appropriateness of our estimated parameter (*L*) through the posterior *predictive* distribution:
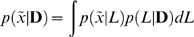
Under the Bayesian model fit, 2/54≈4% of the data falls out of the 95% region. While the nominal error rate is 5%, the actual error rate ≈4% is well within reasonable limits. While the modeling falls under the N-T framework, we've adopted a Bayesian methodology, which provided us with more robust estimates that do not depend on the large sample assumptions in the original paper.

### Analysis of the genomic landscapes of the chromosomal arms in *An. gambiae*

We analyzed the *An. gambiae* AgamP3 genome assembly. Counts and length of coverage of all molecular features were identified in 5-Mb intervals in euchromatin and <1-Mb intervals in heterochromatin. Gene density and transposable element content were analyzed using the Biomart [64] and RepeatMasker (http://www.repeatmasker.org/) programs, respectively. Micro- and minisatellites were analyzed by Tandem Repeats Finder [65]. Only repeats with 80% matches and a copy number of 2 or more (8 or more for microsatellites) were included in the analysis. Microsatellites, minisatellites, and satellites had period size from 2 to 6, from 7 to 99, and from 100 or more, respectively. SDs were detected using BLAST-based whole-genome assembly comparison [66] limited to putative SDs represented by pairwise alignments with ≤2.5-kb and >90 sequence identity. The alignment length was specifically chosen to avoid the vast majority of incompletely masked repetitive elements. SD counts are not discrete duplication events but indicate the number of regions that have been involved in duplications within our interval of interest. Putative MARs in the *An. gambiae* genome sequence were predicted using the SMARTest bioinformatic tool [67]. In order to compare and discern the genome landscape between chromosome arms, we have developed a Generailized Linear Model (GLM) to analyze specified molecular features. We incorporate data that distinguishes both the counts for each molecular feature, and the overall coverage of each feature, in subdivided regions, for each of the five chromosome arms: 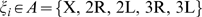. By independence of each region, the likelihood follows as:
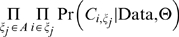
where 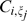 are the counts associated with arm 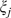, in region *i*. 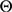 are unknown model parameters that must be estimated. For our application, we used a Poisson random effects model for explaining the counts, but include information about the coverage in each region as well. To make this connection, we parameterize the mean effect, 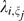, through the canonical log-link function:
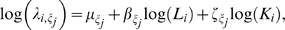
where 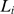 is the total length and 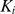 is the coverage length for region *i*.


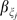
 and 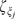 are random effects relating to each of the arm specific lengths. 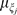 defines the overall density of counts, on each arm. The model unknowns are 
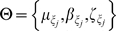
, for each 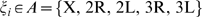. Our goal was to determine if the arm effects: 
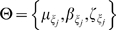
 can be distinguish across arms. Many methods have been proposed for performing such an analysis. Dominant model selection procedures have the ability to compare all possible competing models, and also compensate for the number of parameters involved in each model. That is, if model fit is the objective, then all procedures will determine optimality by utilizing as many parameters as is possible. In our case, these would correspond to 15 possible parameters. Since models selected this way are generally sub-optimal in terms of prediction, likelihood penalization schemes are common practice. For instance, BIC and AIC are commonly used devices for selecting between models. In accordance with these procedures, we select between parsimonious models by maximizing the posterior distribution for each possible model configuration. Automatic multiplicity correction was achieved by penalizing through the prior structure. For our purposes, all prior distribution have been chosen to have the form 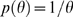, which will achieve the desired results.

As a final step in selecting models, we search through the Maximum *A Posteriori* (MAP) space, associated with each model. We used a simulated annealing algorithm for performing both the model search, and associated parameter maximization. Models with high posterior probability are compared through the ratio: 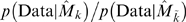, where 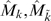 correspond to the MAP models found by the optimization procedure. Table S5 shows mean and median densities and length of coverage as well as mean ranks for all molecular elements in chromosomal arms of *An. gambiae*.

### Analysis of AT/GC content

AT/GC content was calculated using 100-kb nonoverlapping windows with the help of the program ATcontent (Tu 2001). The analysis of AT content was based on a Poisson regression model, since the data arises as discrete counts. Under such a model, the probability of observing the feature count 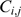, for the 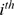 region on chromosome 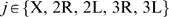, is
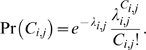
The unknown parameter, 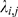 denotes the mean count for observation *i*, on chromosome *j*. This mean form is generalizable to account for different sources of variability found in the data; and in our case, we must account for the variability specific to each chromosome arm 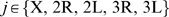, and the length of each region (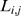). We used the canonical log-link for representing the mean response 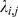 as:
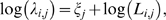
where 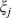 is a chromosome specific random effect for the data.

Since 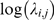 models the logged expectation of counts for each molecular feature, we interpret the estimated parameters by noting the relationship
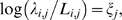
From this, we see that 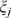 models the AT percent content on chromosome *j*.

While a simple descriptive statistic can be formed for comparing the AT content, across chromosomal arms, such a model based formulation accurately describes the level of variability across the individual arms.

### GO annotation of chromosomal arms

We analyzed the *An. gambiae AgamP4* annotated peptide set using a locally installed copy of Interproscan 4.4.1 [68]. A GO [37] annotation file was generated using Interproscan-assigned GO terms and custom Perl scripts. We used Go-Term-Finder [69] version 0.86 to search for significantly overrepresented (i.e. p<0.05) GO terms assigned to genes in chromosomal arms relative to frequencies for all GO-annotated genes in the peptide dataset. Bar graphs were generated with Microsoft Excel and labeled using Adobe Illustrator CS4.

## Supporting Information

Figure S1 The GRIMM scenario of gene order transformation between the An. gambiae 2R arm and the An. stephensi 2R arm. Relative position and orientation of the conserved syntenic blocks (CSBs) and markers physically mapped to polytene chromosomes are indicated by colored blocks. Numbers over brackets indicate inversion steps. The telomere ends are on the left.
(8.33 MB TIF)

Figure S2 The GRIMM scenario of gene order transformation between the An. gambiae 2L arm and the An. stephensi 3L arm. Relative position and orientation of the CSBs and markers physically mapped to polytene chromosomes are indicated by colored blocks. Numbers over brackets indicate inversion steps. The telomere ends are on the right.
(10.24 MB TIF)

Figure S3 The contrasting patterns of the X chromosome and autosome evolution. The fastest evolution of the X chromosome and parallelism between the extent of inversion polymorphism and inversion fixation rates on the autosomes are shown. The number of fixed inversions (Y axis) is calculated per 1 Mb from GRIMM analysis (the blue bar). The number of all polymorphic inversions in An. gambiae and An. funestus is combined and calculated per 3 Mb (the green bar).
(4.03 MB TIF)

Figure S4 A model of interaction of the 2R and 3L arms with the nuclear envelope. The higher coverage of MARs on 3L generates multiple attachments of this arm to the nuclear envelope. These attachments make more difficult rejoining different breaks and forming inversions despite the abundance of TEs and simple repeats on 3L. In contrast, the lower coverage of MARs on 2R makes fewer nuclear envelope-chromosome contacts and allows more interaction between loci.
(4.36 MB TIF)

Table S1 Physically and in silico mapped DNA markers in the An. gambiae, An. funestus, and An. stephensi genomes.
(0.47 MB DOC)

Table S2 Measures of uniformity of marker distribution for An. gambiae, An. stephensi, and An. funestus.
(0.06 MB DOC)

Table S3 Inversion fixation rates between An. funestus and An. gambaie calculated by GRIMM from the gene order.
(0.05 MB DOC)

Table S4 Posterior estimates for the mean length of each conserved segment (L, Mb) for each of the chromosome arms and the whole genome.
(0.05 MB DOC)

Table S5 Density and coverage of molecular elements in chromosomal arms of An. gambiae.
(0.08 MB DOC)
