# Supplementary figures and images for: Genome Landscape and Evolutionary Plasticity of Chromosomes in Malaria Mosquitoes
Source: PLoS One. 2010 May 12;5(5):e10592. doi: 10.1371/journal.pone.0010592 (PMC2868863; doi:10.1371/journal.pone.0010592)

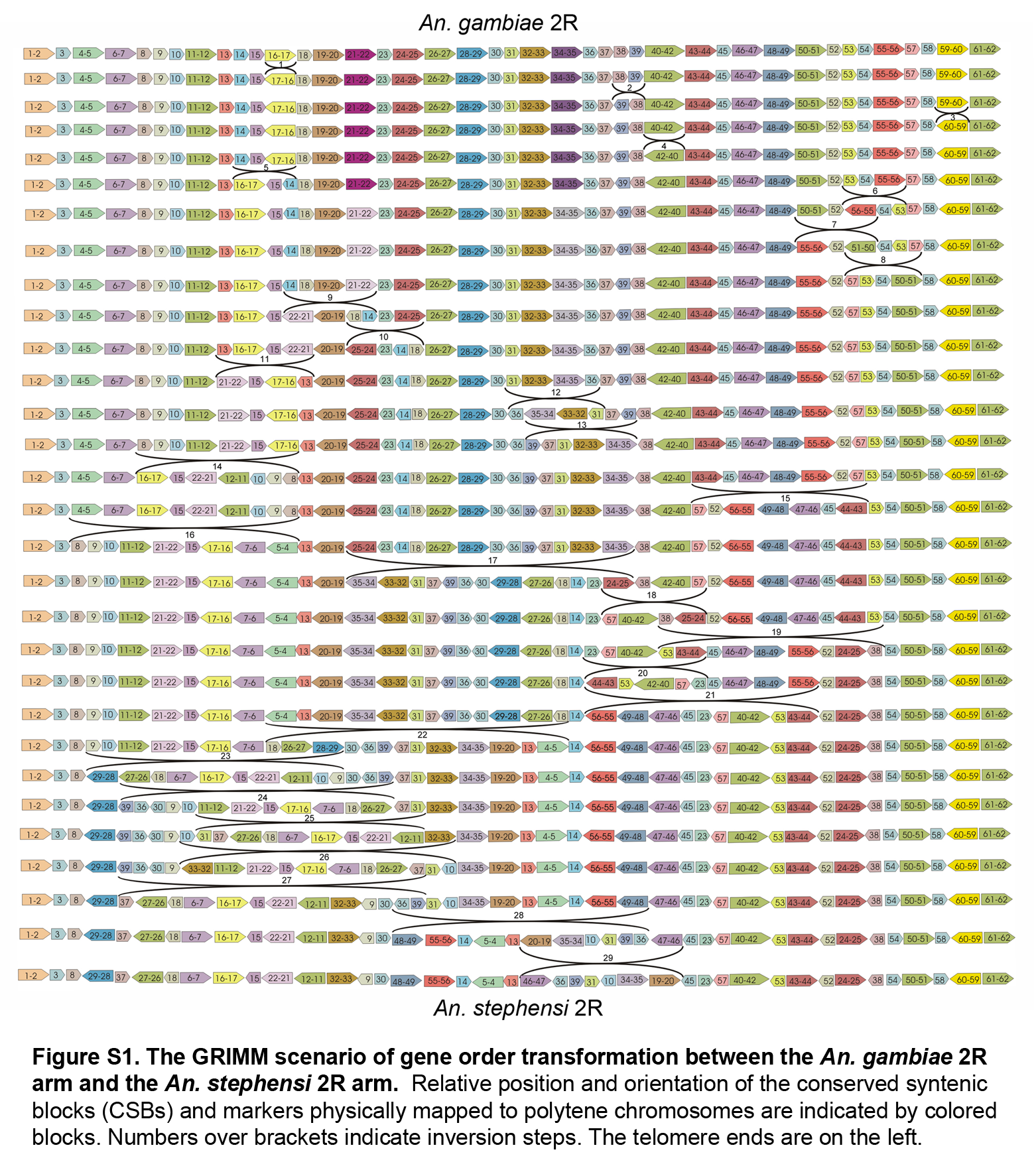

Supplement: Figure S1 — The GRIMM scenario of gene order transformation between the An. gambiae 2R arm and the An. stephensi 2R arm. Relative position and orientation of the conserved syntenic blocks (CSBs) and markers physically mapped to polytene chromosomes are indicated by colored blocks. Numbers over brackets indicate inversion steps. The telomere ends are on the left. (8.33 MB TIF) [file pone.0010592.s001.tif]

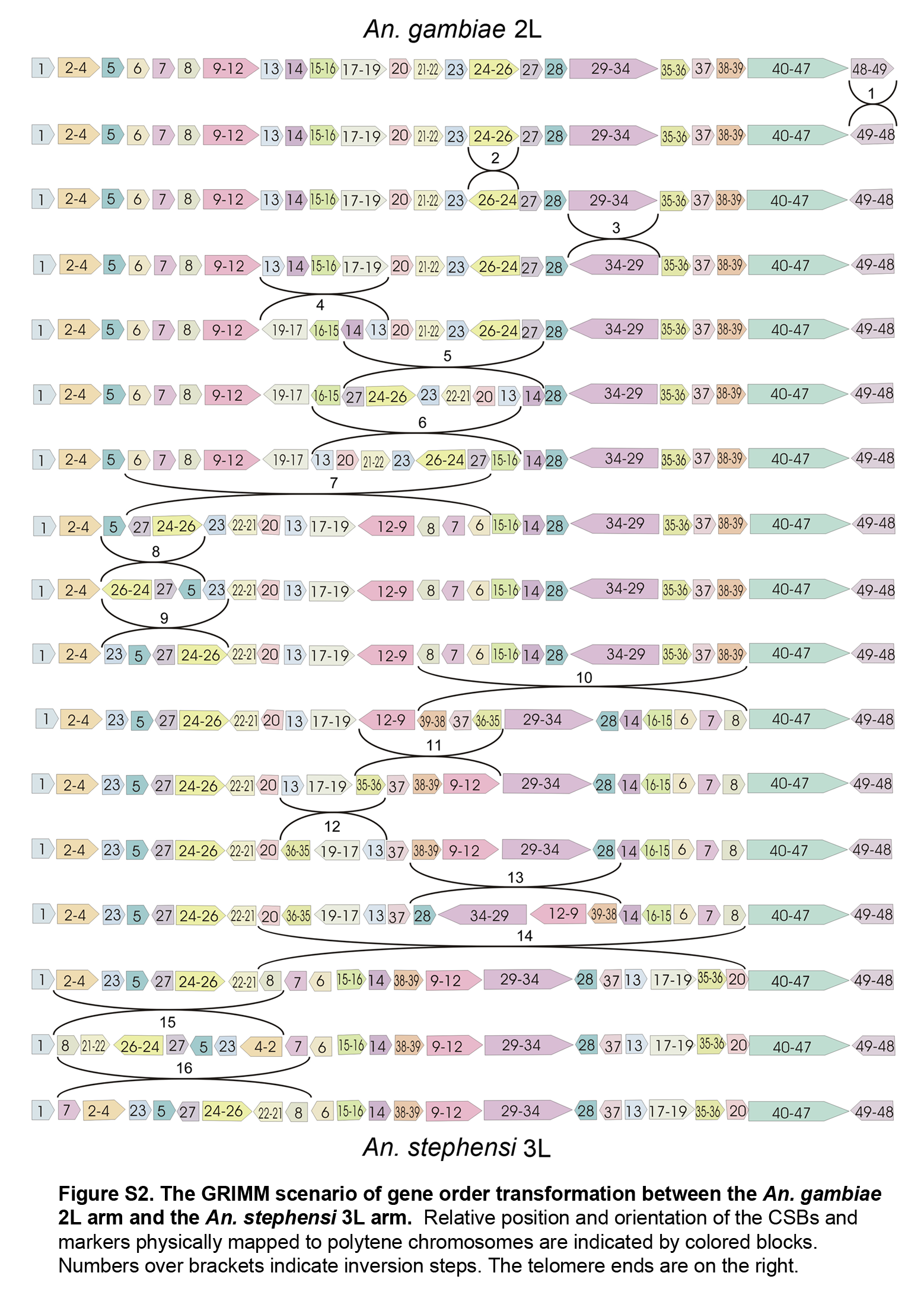

Supplement: Figure S2 — The GRIMM scenario of gene order transformation between the An. gambiae 2L arm and the An. stephensi 3L arm. Relative position and orientation of the CSBs and markers physically mapped to polytene chromosomes are indicated by colored blocks. Numbers over brackets indicate inversion steps. The telomere ends are on the right. (10.24 MB TIF) [file pone.0010592.s002.tif]

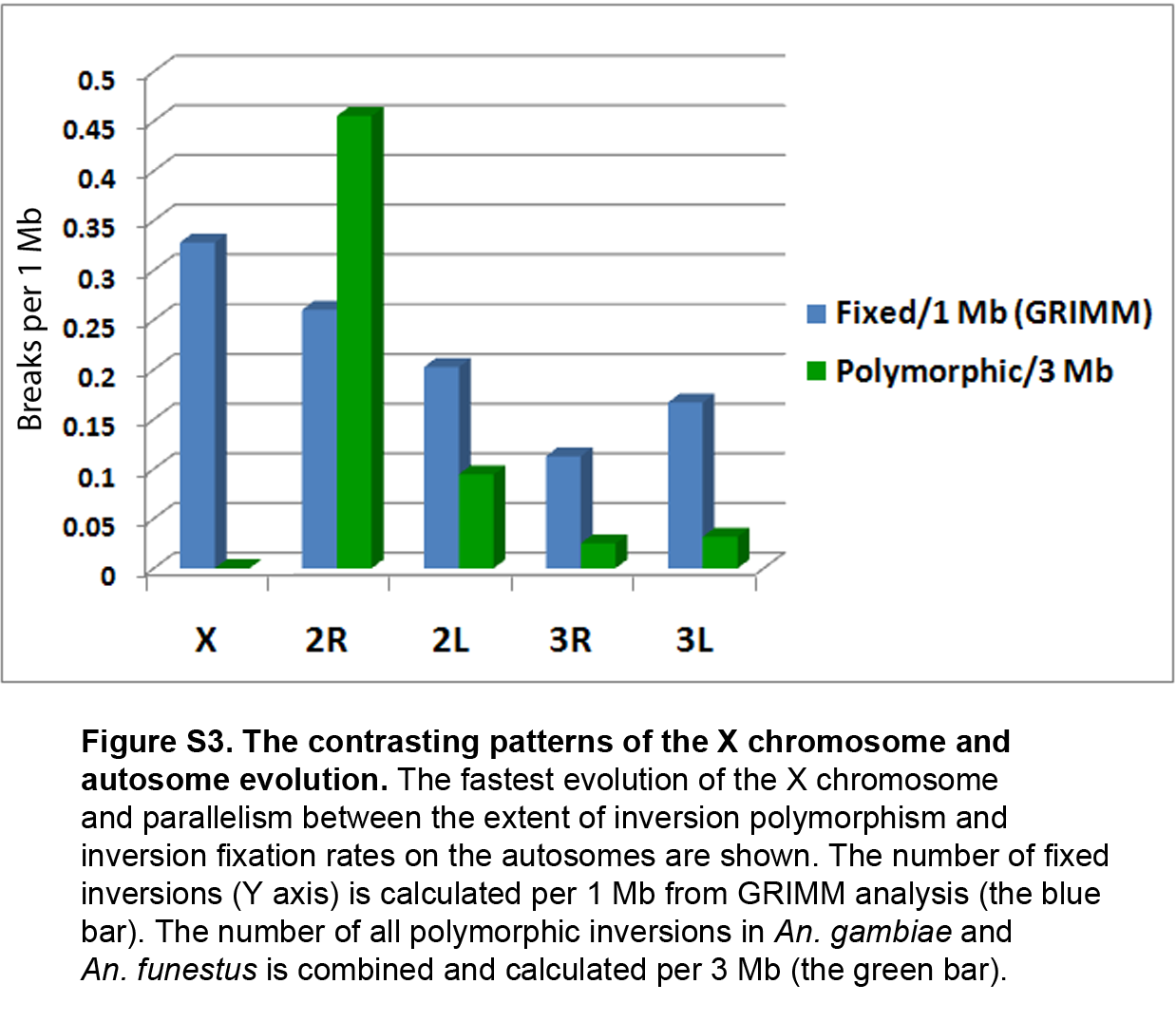

Supplement: Figure S3 — The contrasting patterns of the X chromosome and autosome evolution. The fastest evolution of the X chromosome and parallelism between the extent of inversion polymorphism and inversion fixation rates on the autosomes are shown. The number of fixed inversions (Y axis) is calculated per 1 Mb from GRIMM analysis (the blue bar). The number of all polymorphic inversions in An. gambiae and An. funestus is combined and calculated per 3 Mb (the green bar). (4.03 MB TIF) [file pone.0010592.s003.tif]

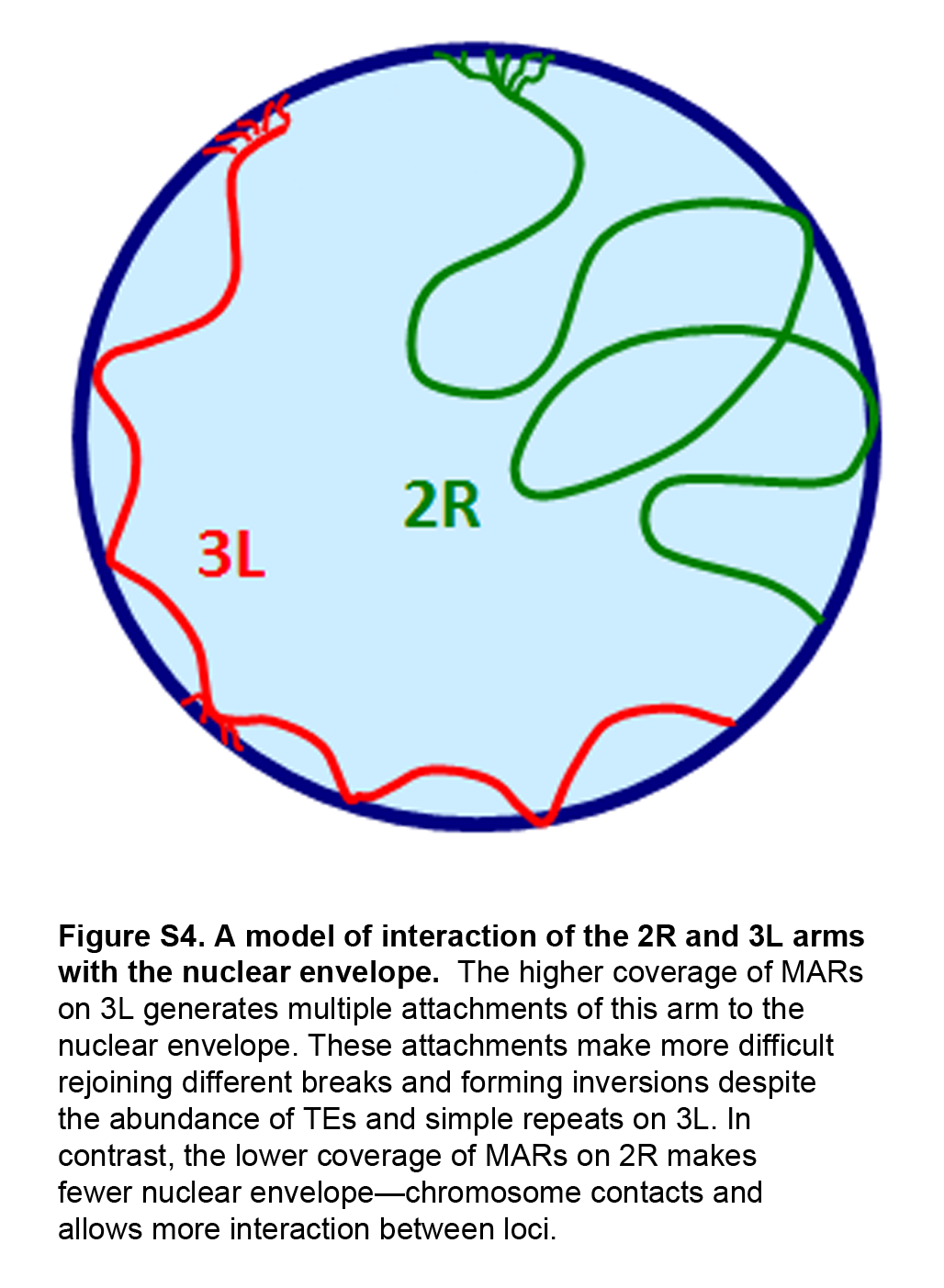

Supplement: Figure S4 — A model of interaction of the 2R and 3L arms with the nuclear envelope. The higher coverage of MARs on 3L generates multiple attachments of this arm to the nuclear envelope. These attachments make more difficult rejoining different breaks and forming inversions despite the abundance of TEs and simple repeats on 3L. In contrast, the lower coverage of MARs on 2R makes fewer nuclear envelope-chromosome contacts and allows more interaction between loci. (4.36 MB TIF) [file pone.0010592.s004.tif]
