# Supplementary material for: Genome Landscape and Evolutionary Plasticity of Chromosomes in Malaria Mosquitoes
Source: PLoS One. 2010 May 12;5(5):e10592. doi: 10.1371/journal.pone.0010592 (PMC2868863; doi:10.1371/journal.pone.0010592)
Supplement: Table S1 — Physically and in silico mapped DNA markers in the An. gambiae, An. funestus, and An. stephensi genomes. (0.47 MB DOC) [file pone.0010592.s005.doc]

**Table S1. Physically and *in silico* mapped DNA markers in the *An. gambiae*, *An. funestus*, and *An. stephensi* genomes**

| Markers on the X chromosome | | | | | | | | | |
| --- | --- | --- | --- | --- | --- | --- | --- | --- | --- |
| Marker  in *An. stephensi* | Markers in *An. funestus* | Clone  name | Accession | VectorBase Gene ID | Genomic Location in  *An. gambiae* | e-value | Chromosomal location | | |
|  |  |  |  |  |  |  | *An. gambiae* | *An. funestus* | *An. stephensi* |
|  | 1 | 06_E11 | BU038887 | AGAP000007 | 84,525-88,380 | 1e-92 | X:4C | X:3D*;  2R:18C | X:3A*;  2R:14B |
| 1 |  | 25E24 | AL610701 |  | 459,415-587,625 | 0 | X:4C | nd | X:5A |
| 2 |  | 126O17 | BH404578 |  | 1,397,208-1,512,610 | 0 | X:4C | nd | X:5A |
|  | 2 | FUN Q | AY116021 | AGAP000087 | 1,451,178-1,454,861 | 7e-03 | X:4C | X:5CD | nd |
| 3 |  | 20N08 | AL150407 |  | 2,363,189-2,453,423 | 0 | X:4A | nd | X:1A |
| 4 |  | 27D16 | AL154011 |  | 2,898,136-3,033,190 | 0 | X:4A | nd | X:1C |
| 5 |  | 150E4 | BH388828 |  | 2,986,103-3,033,400 | 0 | X:4A | nd | X:1C |
|  | 3 | 14_B05 | BU038921 | AGAP000180 | 3,000,117-3,001,769 | 1e-101 | X:4A | X:3A |  |
| 6 |  | 17I02 | AL148591 |  | 3,147,527-3,274,341 | 0 | X:4A | nd | X:1B |
| 7 |  | 24J01 | AL152501 |  | 3,632,054-3,771,323 | 0 | X:3D | nd | X:4A*;  2R:11D; 3R:37B |
| 8 |  | 31G14 | AL156439 |  | 3,944,341-4,029,848 | 0 | X:3D | nd | X:4A |
| 9 |  | 19N19 | AL609649 |  | 5,014,419-5,147,712 | 0 | X:3C | nd | X:1C |
| 10 |  | 26C03 | AL153390 |  | 5,566,114-5,672,612 | 0 | X:3C | nd | X:1C |
| 11 |  | 28F08 | AL154661 |  | 6,001,831-6,002,087 | 0 | X:3B | nd | X:3A |
| 12 |  | 21P07 | AL151067 |  | 7,585,473-7,691,519 | 0 | X:2C | nd | X:3C |
| 13 |  | 17O15 | AL609326 |  | 8,365,624-8,366,297 | 0 | X:2B | nd | X:1A |
| 14 |  | 155N1 | BH384248 |  | 9,048,469-9,170,197 | 0 | X:2B | nd | X:2A |
| 15 |  | 26H02 | AL610921 |  | 11,106,310-11,234,911 | 0 | X:1D | nd |  |
| 16 | 4 | 18_G03 | BU038942 | AGAP000679 | 12,117,478-12,119,325 | 3e-65 | X:1C | X:1B*;  2R:12C;  3L:41C | X:4B |
| 17 |  | 28J20 | AL154814 |  | 13,022,001-13,149,832 | 0 | X:1C | nd | X:2C |
| 18 |  | 24K09 | AL152559 |  | 13,406,025-13,503,738 | 0 | X:1A | nd | X:3B |
| 19 |  | 21F12 | AL150696 |  | 14,068,002-14,190,611 | 0 | X:1B | nd | X:1A |
| 20 |  | 31A03 | AL611577 |  | 14,227,297-14,355,594 | 0 | X:1B | nd | X:3C |
|  | 5 | 17_E02 | BU038934 | AGAP000794  AGAP006891 | 14,442,142-14,443,554  39,426,797-39,428,212 | 1e-137  2e-75 | X:1B*;  2L:26A | X:1C | nd |
| 21 |  | 24G07 | AL152397 |  | 14,548,696-14,659,022 | 0 | X:1B | nd | X:3B |
| 22 |  | Ag0803 | XM_311343 | AGAP000803 | 14,738,909-14,744,472 | 0 | X:5A | nd | X:3B |
|  | 6 | CYP9K1 | AY987362 | AGAP000818 | 15,240,763-15,242,715 | 1e-09 | X:5A | X:5B | nd |
| 23 |  | 13E12 | AL146915 |  | 15,322,088-15,435,208 | 0 | X:5A | nd | X:4A |
|  | 7 | 61_C09 | BU039001 | AGAP000824 | 15,336,624-15,339,506 | 1e-33 | X:5A | X:2B | nd |
|  | 8 | FUN E | AY116009 |  | 15,736,855-15,736,883  23,418,653-23,418,681 | 9e-02 | X:5A | X:5D | nd |
|  | 9 | CYP4G21 | AY648704 | AGAP000877 | 16,619,141-16,621,043 | 4e-32 | X:5AB | X:4C | nd |
| 24 |  | 138A5 | BH380601 |  | 16,677,135-16,799,196 | 0 | X:5B | nd | X:1C |
|  | 10 | 98_D11 | BU039017 | AGAP000886 | 16,737,992-16,741,889 | 1e-14 | X:5B | X:1C | nd |
|  | 11 | 26_G11 | BU038979 | AGAP000953  AGAP002395 | 18,339,741-18,342,792  20,904,493-20,905,615 | 1e-102  7e-78 | X:5C*;  2R:12B | X:1B*;  2L:28C | X:4B |
| 25 | 12 | 18_D02 | BU038939 | AGAP001036 | 19,936,008-19,937,213 | 2e-87 | X:6 | X:3D | X:1B |
|  | 13 | 18_B08 | BU038937 | AGAP001076 | 22,937,938-22,947,129 | 1e-60 | X:6 | X:6 | nd |
| Markers on 2R | | | | | | | | | |
| Markers in *An. stephensi* | Markers in *An. funestus* | Clone  name | Accession | VectorBase Gene ID | Genomic Location in *An. gambiae* | e-value | Chromosomal location | | |
|  |  |  |  |  |  |  | *An. gambiae* | *An. funestus* | *An. stephensi* |
| 1 |  | 155B23 | BH368691 |  | 528,525-648,904 | 0 | 2R:7A | nd | 2R:7A |
|  |  | 23_G01 | BU038967 | AGAP001141 | 582,418-585,357 | 2e-34 | 2R:7A | 2R:10C5*,17B | nd |
|  |  | 01_D07 | BU038871 | AGAP001164 | 673,798-674,927 | 1e-174 | 2R:7A | 2R:10C*,7C | nd |
| 2 |  | 04L11 | AL141975 |  | 1,599,882-1,708,302 | 0 | 2R:7B | nd | 2R:7A |
|  | 1 | 21_F03 | BU038956 | AGAP001215 | 1,608,204-1,609,027 | 1e-23 | 2R:7B | 2R:7B | nd |
| 3 | 2 | 01_H04 | BU038873 | AGAP001306 | 2,802,248-2,803,419 | 1e-165 | 2R:7B | 2R:7A | 2R:7B |
| 4 | 3 | 21_F12 | BU038958 | AGAP001380 | 4,050,701-4,051,820 | 2e-69 | 2R:8A | 2R:12D | 2R:13A |
|  |  | 07_E10 | BU038891 | AGAP001394 | 4,153,647-4,167,690 | 3e-49 | 2R:8A | 2R:12D | nd |
| 5 |  | 140D21 | BH370864 |  | 4,677,078-4,755,258 | 0 | 2R:8A | nd | 2R:12D |
|  | 4 | 06_E01 | BU038885 | AGAP001420 | 4,739,226-4,740,361 | 1e-162 | 2R:8A | 2R:12E | nd |
| 6 |  | AsOBP-7 | EU816361 | AGAP001556 | 6,152,011-6,168,838 | 1e-38 | 2R:8C | nd | 2R:9C |
|  |  | 36_B06 | BU038996 | AGAP001588  AGAP012818 | 6,491,921-6,495,261  31,394,884-31,395,776 | 3e-80  1e-82 | 2R:8C;  UNKN | 2R:8E | nd |
| 7 |  | 153L12 | BH380684 |  | 6,813,959-6,894,483 | 0 | 2R:8D | nd | 2R:9C |
|  | 5 | 36_E01 | BU039000 | AGAP001617 | 6,827,869-6,831,707 | 1e-75 | 2R:8D | 2R:8D | nd |
| 8 | 6 | 12_G10 | BU038913 | AGAP001721 | 8,925,685-8,926,882 | 1e-105 | 2R:8E | 2R:15C | 2R:8A |
|  |  | 16_A10 | BU038927 | AGAP001734 | 9,093,431-9,095,845 | 2e-22 | 2R:8E | 2R:15C*;  2L:25D | 2R:7A |
| 9 |  | Ag1759 | XM_321324 | AGAP001759 | 9,479,549-9,483,291 | 0 | 2R:8E | nd | 2R:11A |
| 10 |  | Ag1763 | XM_321320 | AGAP001763 | 9,523,856-9,528,904 | 0 | 2R:9A | nd | 2R:16A |
| 11 |  | Ag1780 | XM_321295 | AGAP001780 | 10,216,796-10,230,335 | 0 | 2R:9A | nd | 2R:16A |
| 12 |  | 25P09 | AL153306 |  | 10,530,256-10,650,334 | 0 | 2R:9A | nd | 2R:10D |
|  | 7 | AFND5 | AF171035 | AGAP001797 | 10,545,856-10,583,613 | 5.5e-18 | 2R:9A | 15B | nd |
| 13 |  | 11A13 | AL145719 |  | 11,505,616-11,604,069 | 0 | 2R:9B | nd | 2R:14B |
|  |  | FUN O | AY116019 |  | 11,565,685-11,565,728 | 3e-05 | 2R:9B | 2R:18A | nd |
|  | 8 | 23_B02 | BU038961 | **AGAP001903** | 11,975,829-11,977,395 | 1e-111 | 2R:9B | 2R:18B | nd |
| 14 |  | 20N10 | AL150410 |  | 12,874,429-12,976,648 | 0 | 2R:9C | nd | 2R:12C |
| 15 |  | Ag1980 | XM_321082 | **AGAP001980** | 13,087,584-13,091,963 | 0 | 2R:9C | nd | 2R:10A |
|  | 9 | 36_B02 | BU038995 | AGAP001983 | 13,133,425-13,135,252 | 1e-48 | 2R:9C | 2R:9B | nd |
| 16 |  | Ag2015 | XM_321040 | AGAP002015 | 13,936,706-13,955,790 | 0 | 2R:10A | nd | 2R:9D |
|  | 10 | 11_D03 | BU038903 | AGAP002020  AGAP012731 | 14,012,136-14,013,179  25,798,127-25,799,580 | 4.5e-118  5.2e-59 | 2R:10A  UNKN | 2R:9A | 2R:10A |
| 17 |  | 22D14 | AL151203 |  | 14034511-14034752 | 0 | 2R:10A | nd | 2R:10A |
| 18 |  | 135P16 | BH387168 |  | 14,892,477-14,952,998 | 0 | 2R:10B | nd | 2R:9B |
| 19 | 11 | 04_D06 | BU038877 | AGAP002166 | 16,812,718-16,815,833 | 6e-35 | 2R:11A | 2R:10C | 2R:16AB |
| 20 |  | 137K7 | BH371689 |  | 17,597,143-17,688,592 | 0 | 2R:11A | nd | 2R:16C |
|  | 12 | 61_D05 | BU039002 | AGAP002213 | 17,648,143-17,653,542 | 3e-91 | 2R:11A | 2R:10C | nd |
| 21 | 13 | 08_E06 | BU038895 | AGAP002317 | 19,444,433-19,447,708 | 4e-61 | 2R:11C | 2R:16A | 2R:10D |
| 22 |  | AF13C05 | F.H. Collins lab |  | 19,883,222-19,883,271 | 4e-17 | 2R:11C | 2R:16A | 2R:10C |
|  | 14 | 06_D06 | BU038884 |  | 19,946,249-19,946,745 | 1e-42 | 2R:11C | 2R:16B | nd |
| 23 |  | 29F01 | AL155230 |  | 20,468,607-20,555,519 | 0 | 2R:11C | nd | 2R:17A |
|  | 15 | 15_G03 | BU038926 | AGAP002413 | 21,027,264-21,030,999 | 6e-75 | 2R:12A | 2R:12A | nd |
| 24 |  | 25_E09 | BU038972 | AGAP002440 | 21,425,189-21,426,085 | 3e-83 | 2R:12B | 2R:12B | 2R:18B |
|  |  | 129M18 | BH377340 |  | 21,521,726-21,621,799 | 0 | 2R:12B |  | 2R:18B |
|  | 16 | 13_F11 | BU038919 | AGAP002457 | 21,573,636-21,587,145 | 6e-57 | 2R:12B | 2R:12B2 |  |
|  |  | 626240 | BM655548 | AGAP002465 | 21,825,430-21,827,282 | 1e-136 | 2R:12B | nd | 2R:18B |
|  | 17 | 15_F10 | BU038925 | AGAP002465 | 21,825,430-21,827,282 | 9e-65 | 2R:12B | 2R:12B | nd |
| 25 | 18 | 17_G08 | BU038935 | AGAP002468 | 21,835,973-21,836,716 | 1e-153 | 2R: 12B | 2R:12B;  2L:23C | 2R:18C |
| 26 |  | AsGPROR7 | Z. Tu lab | AGAP002560 | 22,849,252-22,858,650 | 1e-159 | 2R: 12C | nd | 2R:9A |
| 27 | 19 | 06_B01 | BU038882 | AGAP002606 | 23,835,495-23,836,568 | 1e-90 | 2R:12C | 2R:14D | 2R:9A |
|  |  | 20_E04 | BU038952 | AGAP002608 | 23,842,548-23,842,828 | 1e-27 | 2R:12C | 2R:14D | nd |
| 28 |  | 09N07 | AL145079 |  | 25,146,360-25,266,886 | 0 | 2R:12D | nd | 2R:8C |
| 29 |  | 18L04 | AL149296 |  | 26,187,553-26,313,071 | 0 | 2R:12E | nd | 2R:8B |
| 30 |  | 155H21 | BH398459 |  | 27,025,144-27,125,121 | 0 | 2R:13A |  | 2R:11B |
|  | 20 | AFND32 | AY291371 |  | 27,025,145-27,125,121 | 0.040 | 2R:13A | 2R:15E | nd |
|  |  | AFND37 | AY291373 | AGAP002790 | 27,262,932-27,311,956 | 1e-24 | 2R:13A | 2R:15E | nd |
| 31 |  | 138H21 | BH381119 |  | 28,310,572-28,311,109 | 0 | 2R:13C | nd | 2R:15B |
| 32 |  | AsRPS6 | AY237124 | AGAP002919 | 29,609,284-29,611,282 | 0 | 2R:13C | nd | 2R:11A |
| 33 |  | Ag2934 | XM_001237408 | AGAP002934 | 29,835,568-29,836,999 | 0 | 2R: 13C | nd | 2R:11A |
| 34 |  | Ag2935 | XM_311967 | AGAP002935 | 29,839,387-29,840,621 | 0 | 2R:13C | nd | 2R:15B |
|  | 21 | 25_H11 | BU038976 | AGAP002935 | 29,839,388-29,840,621 | 8e-49 | 2R: 13C | 2R:10B | nd |
|  |  | 01C03 | AL139911 |  | 29,974,464-30,077,977 | 0 | 2R:13D | nd | 2R:15B |
| 35 |  | 27I24 | AL154218 |  | 30,150,936-30,271,431 | 0 | 2R:13D | nd | 2R:16A |
| 36 |  | 139N4 | BH379254 |  | 30,694,330-30,796,747 | 0 | 2R:13D | nd | 2R:14C |
|  |  | 66_G12 | BU039012 | AGAP002994 | 30,716,522-30,717,395 | 2e-75 | 2R, 13D | 2R, 17C*; 3R, 34A | nd |
| 37 |  | 31M01 | AL611707 |  | 31,181,535-31,317,988 | 0 | 2R:13E | nd | 2R:8C |
|  | 22 | 03_D09 | BU038874 | AGAP003024 | 31,230,560-31,232,558 | 5e-27 | 2R:13E | 2R:17C | nd |
| 38 |  | 09E12 | AL144757 |  | 33,575,891-33,693,463 | 0 | 2R:14C | nd | 2R:18C |
|  |  | 23_C09 | BU038963 | AGAP003184 | 33,620,457-33,622,184 | 1e-82 | 2R, 14C | X, 4C 2R, 12B* | nd |
| 39 | 23 | 11_D07 | BU038904 | AGAP003209 | 33,903,940-33,905,170 | 1e-143 | 2R:14C | 2R:13A | 2R:15A |
| 40 |  | 21I20 | AL609973 |  | 34,742,360-34,873,652 | 0 | 2R:14D | nd | 2R:17A |
| 41 |  | AsOBP1 | Z. Tu lab | AGAP003309 | 35,643,035-35,644,609 | 1e-89 | 2R:14D | nd | 2R:17A |
| 42 |  | 23O01 | AL152140 |  | 35,770,783-35,790,690 | 0 | 2R:14E | nd | 2R:17A |
|  |  | 22_H10 | BU038960 | AGAP003312 | 35,775,371-35,775,597 | 1e-51 | 2R:14E | 2R:13B | nd |
| 43 |  | Ag3315 | XM_319546 | AGAP003315 | 35,837,690-35,839,243 | 0 | 2R:15A | nd | 2R:17B |
| 44 |  | Ag3342 | XM_314239 | AGAP003342 | 36,307,756-36,311,720 | 0 | 2R:15A | nd | 2R:17C |
| 45 |  | Ag3351 | XM_001688498 | AGAP003351 | 36478446-36480531 | 0 | 2R:15A | nd | 2R:16C |
| 46 |  | Ag3363 | XM_314265 | AGAP003363 | 36,850,789-36,853,077 | 0 | 2R:15A | nd | 2R:14A |
|  | 24 | 27_E05 | BU038983 | AGAP003384 | 37,194,861-37,195,629 | 1e-54 | 2R:15B | 2R:18C | nd |
|  | 25 | 12_G11 | BU038914 | AGAP003416 | 37,466,896-37,468,119 | 3e-40 | 2R:15BC | 2R:18C | nd |
| 47 |  | Ag3434 | XM_311720 | AGAP003434 | 37,703,243-37,711,020 | 0 | 2R:15BC | nd | 2R:14B |
| 48 | 26 | 12_H09 | BU038915 | AGAP003500 | 38,738,669-38,739,898 | 5e-82 | 2R:15C | 2R:18D | 2R:11C |
|  | 27 | 66_E07 | BU039009 | AGAP003553 | 39,452,027-39,453,046 | 7e-44 | 2R:15D | 2R:16C | nd |
| 49 | 28 | AF261B04 | F.H. Collins lab |  | 39,751,219-39,751,269 | 2e-15 | 2R:15D | 2R:16C | 2R:11D |
|  |  | 66_A02 | BU039006 | AGAP003625 | 40,683,416-40,683,582 | 1e-37 | 2R:15E | 2R:12C | nd |
| 50 |  | 169F11 | BH369697 |  | 40,944,146-41,044,921 | 0 | 2R:15E | nd | 2R:19A |
|  | 29 | 29_F03 | BU038988 | AGAP003650  AGAP012611 | 41,002,243-41,003,465  19,943,725-19,944,865 | 5e-48  1e-45 | 2R:15E;  UNKN | 2R:11B | nd |
| 51 | 30 | 11_E07 | BU038905 | AGAP003664 | 41,338,854-41,360,919 | 1e-131 | 2R:16A | 2R:14C | 2R:19A |
|  |  | 36_A12 | BU038994 | AGAP003790 | 43,342,500-43,352,336 | 0 | 2R:16B | 2R:8C*;  3R:35E | nd |
| 52 |  | 142O19 | BH368703 |  | 45,428,742-45,560,792 | 0 | 2R:16D | nd | 2R:18A |
|  | 31 | 13_A06 | BU038916 | TCLAG158671 | 45,474,529-45,474,606  20,332,357-20,332,644 | 2e-31  2e-31 | 2R:16D;  UNKN | 2R:13D | nd |
| 53 |  | 08O05 | AL144514 |  | 47,277,152-47,354,855 | 0 | 2R:17B | nd | 2R:17A |
|  | 32 | 13_C03 | BU038918 | AGAP003971 | 47,284,629-47,308,582 | 3e-55 | 2R:17B | 2R:13C | nd |
| 54 |  | 157B8 | BH384608 |  | 48,013,258-48,137,831 | 0 | 2R:17C | nd | 2R:18D |
|  |  | 20_A10 | BU038950 | AGAP004085 | 49,526,117-49,527,342 | 5e-45 | 2R:17C | 2R:16C*, 18C1, 18C4;  2L:23D, 26A; 3R: 36E; 3L:41A, 42B | nd |
| 55 |  | 166G9 | BH383888 |  | 52,013,039-52,095,531 | 0 | 2R:18B | nd | 2R:12B |
| 56 | 33 | 18_D12 | BU038940 | AGAP004247 | 53,207,213-53,209,347 | 7e-71 | 2R:18C | 2R:14B | 2R:12C |
| 57 |  | AsSP11.9 | AY162245 | AGAP004316 | 54,393,078-54,393,473 | 2e-28 | 2R:18D | nd | 2R:17A |
| 58 |  | 211F02 | EX227558 | AGAP004422 | 55,869,598-55,870,844 | 0 | 2R:19A | nd | 19B |
| 59 |  | 23I15 | AL151968 |  | 56,667,003-56,767,697 | 0 | 2R:19B | nd | 2R:19C |
| 60 | 34 | 11_B04 | BU038900 | AGAP004552 | 57,542,706-57,543,865 | 1e-132 | 2R:19C | 2R:19C | 2R:19BC |
| 61 |  | 17N16 | AL148800 |  | 58,537,340-58,618,071 | 0 | 2R:19D | nd | 2R:19E |
| 62 |  | StBAC62 | Y. Shouche lab | AGAP004662 | 60,236,329-60,253,235 | 1e-89 | 2R:19D | nd | 2R:19E |
| Markers on 2L | | | | | | | | | |
| Markers in *An. stephensi* | Markers in *An. funestus* | Clone  name | Accession | VectorBase Gene ID | Genomic Location in  *An. gambiae* | e-value | Chromosomal location | | |
|  |  |  |  |  |  |  | *An. gambiae* | *An. funestus* | *An. stephensi* |
| 1 |  | AsHyp16 | AY162228 | AGAP004799 | 3,720,848-3,721,878 | 1e-103 | 2L:20D | nd | 3L:38E |
| 2 |  | 101C3 | BH388218 |  | 5,708,924-5,858,418 | 0 | 2L:21A | nd | 3L:38B |
|  | 1 | 28_C07 | BU038985 | AGAP004904 | 5,771,985-5,780,988 | 4e-67 | 2L:21A | 3R:35C | nd |
| 3 |  | 03G12 | AL141218 |  | 6,081,630-6,182,049 | 0 | 2L:21A | nd | 3L:38B |
|  | 2 | 29_H01 | BU038990 | AGAP004929 | 6,114,439-6,115,700 | 2e-22  2e-22 | 2L:21A  UNKN | 3R:35B | nd |
|  | 3 | FUN D | AY116008 |  | 6,734,718-6,734,834 | 1e-34 | 2L:21A | 3R:35B | nd |
| 4 |  | 02A19 | AL140406 |  | 8,667,456 - 8,791,152 | 0 | 2L:21C | nd | 3L:39D |
|  | 4 | 04_D07 | BU038878 | AGAP005037 | 8,668,402-8,700,721 | 8e-30 | 2L:21C | 3R:35A | nd |
| 5 |  | 02A04 | AL140380 |  | 9,902,459- 9,981,236 | 0 | 2L:21D | nd | 3L:39C |
|  | 5 | 25_E12 | BU038973 | AGAP005117 | 10,241,196-10,244,380 | 1e-97 | 2L:21D | 3R:36F | nd |
| 6 |  | 10I04 | AL145436 |  | 11,534,512-11,595,103 | 0 | 2L:21E | nd | 3L:40B |
| 7 |  | 157I18 | BH367786 |  | 13,513,086-13,564,568 | 0 | 2L:21F | nd | 3L:38C |
| 8 |  | 26E08 | AL610881 |  | 14,601,776-14,728,697 |  | 2L:22A | nd | 3L:40C |
| 9 |  | 23_D08 | BU038965 | AGAP005410 | 15,211,390-15,213,382 | 5e-20 | 2L:22B | 3R:35C*,  34A;  2R:16B,19A | 3L:41B*;  2L:25A |
| 10 |  | 03C15 | AL141092 |  | 16,516,545-16,614,317 |  | 2L:22C | nd | 3L:41A |
| 11 |  | 27O10 | AL154432 |  | 17,882,981-17,974,647 | 0 | 2L:22D | nd | 3L:42C |
|  | 6 | 95_H01 | BU039015 | AGAP005618 | 17,920,080-17,921,140 | 4e-27 | 2L:22D | 3R:31D | nd |
| 12 |  | 104C14 | BH391906 |  | 18,512,073-18,616,664 |  | 2L:22E |  | 3L:42C |
|  | 7 | 20_D11 | BU038951 | AGAP005712 | 19,277,265-19,282,704 | 1e-58 | 2L:22E | 3R:33C | nd |
| 13 |  | 131F22 | BH390198 |  | 20,364,135-20,459,325 | 0 | 2L:22F | nd | 3L:44C |
|  | 8 | AFND19 | AF171049 | AGAP005770 | 20,396,064-20,405,726 | 9.9e-84 | 2L:22F | 3R:34A | nd |
| 14 |  | SuaPh6_1.8EcoRI | NA | AGAP005780 | 20,535,740-20,538,254  20,538,082-20,543,536 | 0 | 2L: 23A | nd | 3L:40A |
|  | 9 | 30_G04 | BU038991 | AGAP005862 | 21,199,038-21,200,529 | 1e-133 | 2L: 23A | 3R:33D | nd |
| 15 |  | AsSP53.7 | AY162233 | AGAP005822 | 21,865,434-21,867,120 | 7e-86 | 2L:23B | nd | 3L:40A |
| 16 |  | 716320 | BM606621 | AGAP005838 | 22,321,985-22,327,760 | 0 | 2L:23B | nd | 3L:40A |
|  | 10 | 66_E11 | BU038987 | AGAP005838 | 22,321,985-22,327,760 | 1e-134 | 2L:23B | 3R:33D | nd |
|  |  | AsK3MO | AY065662 | AGAP005948 | 23,985,166-23,988,714 | 0 | 2L:23C | nd | 3L:44B*; 2R:15A,  17C; 3R:35B, 37C |
| 17 |  | 12G16 | AL146467 |  | 24,626,085-24,724,371 | 0 | 2L:23C | nd | 3L:44A |
|  | 11 | 29_E12 | BU039020 | AGAP006015 | 24,671,856-24,674,367 | 4e-36 | 2L:23C | 3R:33A | nd |
| 18 |  | 211A02 | EX227513 | AGAP006037 | 25,521,114-25,523,716 | 1e-105 | 2L:23D | nd | 3L:44A |
| 19 |  | 150F12 | BH385494 |  | 25,924,194-26,087,837 | 0 | 2L:23D | nd | 3L:44A |
|  | 12 | 36_A10 | BU038993 | AGAP006071 | 26,055,839-26,056,556 | 8e-25 | 2L:23D | 3R:35F | nd |
| 20 | 13 | 61_E02 | BU039003 | AGAP006148 | 27,158,330-27,159,179 | 9e-86 | 2L:24A | 3R:30C | 3L:45A |
| 21 |  | AsPPO1 | AY559300 | AGAP006258 | 28,702,474-28,705,612 | 1e-103 | 2L:24B | nd | 3L:40D |
|  | 14 | 18_G09 | BU038943 | AGAP006263 | 28,771,405-28,774,168 | 1e-115 | 2L:24B | 3R:35F | nd |
| 22 |  | 04P13 | AL142126 |  | 29,574,218-29,659,442 | 0 | 2L:24B | nd | 3L:40D |
| 23 |  | 140N16 | BH384642 |  | 30,990,832-31,090,638 | 0 | 2L:24D | nd | 3L:39C |
|  | 15 | 21_E03 | BU038955 | AGAP006389 | 31,048,592-31,050,091 | 2e-35 | 2L:24D | 3R:35F | nd |
| 24 |  | AsAG5 | AY162227 | AGAP006421 | 31,693,742-31,694,830 | 2e-66 | 2L:24D | nd | 3L:39B |
| 25 |  | AST004P4 | Z. Tu lab |  | 31,715,321-31,760,529 | 0 | 2L:24D | nd | 3L:39A |
| 26 |  | 212G03 | EX227637 | AGAP006442 | 32,055,094-32,055,857 | 8E-39 | 2L:24D | nd | 3L:39A |
| 27 |  | 10D09 | AL145285 |  | 32,640,047-32,736,529 | 0 | 2L:25A | nd | 3L:39B |
| 28 |  | 02K19 | AL140790 |  | 35,622,251-35,720,581 | 0 | 2L:25C | nd | 3L:43A |
|  |  | 109B13 | BH385033 |  | 36,603,004-36,704,974 | 0 | 2L:25D | nd | 3L:42B, 44B |
|  | 16 | 95_D09 | BU039013 | AGAP006677 | 36,653,143-36,654,063 | 2e-48 | 2L:25D | 3R:33C | nd |
| 29 |  | 212D03 | EX227607 | AGAP006709 | 37,118,475-37,119,488 | 4e-30 | 2L:25D | nd | 3L:42B |
| 30 |  | 26L15 | AL153718 |  | 37,497,029-37,625,484 | 0 | 2L:25D | nd | 3L:42B |
| 31 |  | 101L14 | BH382930 |  | 38,518,994-38,594,734 | 0 | 2L:26A | nd | 3L:42A |
|  | 17 | 06_C09 | BU038883 | AGAP006795 | 38,567,686-38,568,743 | 13e-14 | 2L:26A | 3R:32B | nd |
| 32 | 18 | 16_F07 | BU038931 | AGAP006861 | 39,215,802-39,216,191 | 1e-129 | 2L:26B | 3R:36E | 3L:42A |
| 33 |  | 211H03 | EX227578 | AGAP006871 | 39,285,040-39,287,251 | 1e-71 | 2L:26B | nd | 3L:42A |
| 34 | 19 | 09_C11 | BU038897 | AGAP006918 | 39,995,907-39,997,107 | 1e-63 | 2L:26C | 3R:36D | 3L:43C |
| 35 | 20 | AF262H10 | F.H. Collins lab | AGAP006975 | 40,442,200-40,442,600 | 8e-55 | 2L:26C | 3R:35F | 3L:45C |
| 36 | 21 | 11_F09 | BU038906 | AGAP006996 | 40,536,371-40,538,248 | 1e-100 | 2L:26D | 3R:35F | 3L:45C |
| 37 |  | Ag7063 | XM_308701 | AGAP007063 | 42,126,193-42,127,751 | 0 | 2L:26D | nd | 3L:44C |
| 38 |  | Ag7070 | XM_001688008 | AGAP007070 | 42,178,250-42,181,793 | 0 | 2L:27A | nd | 3L:40A |
| 39 |  | 31L22 | AL156623 |  | 42,206,288-42,309,500 | 0 | 2L:27A | nd | 3L:40A |
|  |  | AFND33 | AY291372 | AGAP007078 | 42,216,309-42,216,467 | 1e-12 | 2L:27A | 3R:33C | nd |
| 40 |  | 04C08 | AL607764 |  | 43,540,182-43,634,335 | 0 | 2L:27A | nd | 3L:45A |
|  | 22 | 08_B09 | BU038894 | AGAP007160 | 43,603,779-43,607,408 | 1e-48 | 2L:27A | 3R:30C | nd |
| 41 | 23 | 06_G08 | BU038889 | AGAP007249 | 44,638,197-44,642,288 | 5e-56 | 2L:27C | 3R:30C | 3L:46D |
| 42 |  | 178B1 | BH372501 |  | 45,026,766-45,130,891 | 0 | 2L:27C | nd | 3L:46D |
|  |  | 25_D11 | BU039019 | AGAP007297 | 45,066,291-45,067,164 | 1e-116 | 2L:27C | 3R:30B | nd |
| 43 |  | 26_B05 | BU038978 | AGAP007309 | 45,256,867-45,259,724 | 4e-21 | 2L:27C | 3R:31C | 3L:46CD |
|  | 24 | 23_E09 | BU038966 | AGAP007347 | 45,981,005-45,981,721 | 9e-09 | 2L:27D | 3R:30A | nd |
| 44 |  | 26_A01 | BU038977 | AGAP007362 | 46,062,101-46,079,300 | 1e-76 | 2L:27D | 3R:30A | 3L:46C |
| 45 | 25 | 07_A01 | BU038890 | AGAP007406 | 46,337,490-46,341,061 | 8e-58 | 2L:27D | 3R:29D | 3L:46C |
| 46 | 26 | 11_B01 | BU038899 | AGAP007508 | 46,995,122-46,995,883 | 2e-96 | 2L:28A | 3R:29C | 3L:46C |
|  |  | 14_C12 | BU038922 | AGAP007558  AGAP007414 | 47,556,815-47,558,944  46,395,202-46,395,819 | 0  2e-16 | 2L:28B*  2L:28A | 3R:29C | nd |
| 47 |  | 211B01 | EX227521 | AGAP007618 | 48,340,253-48,341,468 | 3e-48 | 2L:28C | nd | 3L:46A |
| 48 | 27 | 18_G01 | BU038941 | AGAP007643 | 48,608,644-48,628,119 | 7e-69 | 2L:28C | 3R:29B | 3L:46A |
| 49 |  | 142L24 | BH399793 |  | 49,243,380-49,333,455 | 0 | 2L:28D | nd | 3L:46A |
| Markers on 3R | | | | | | | | | |
| Markers in *An. stephensi* | Markers in *An. funestus* | Clone  name | Accession | VectorBase Gene ID | Genomic Location in *An. gambiae* | e-value | Chromosomal location | | |
|  |  |  |  |  |  |  | *An. gambiae* | *An. funestus* | *An. stephensi* |
| 1 |  | 212A07 | EX227590 | AGAP007747 | 217,370-218,365 | 4e-22 | 3R:29A | nd | 3R:29C |
| 2 |  | AsSki | AY578814 | AGAP007776 | 620,123-621,199 | 1e-110 | 3R:29A | nd | 3R:29B |
|  |  |  |  |  |  |  |  |  |  |
| 3 | 1 | 19_F10 | BU038947 | AGAP007786 | 838,035-840,304 | 1e-167 | 3R:29A | 2L:27D | 3R:29B |
| 4 |  | 10J02 | AL608684 |  | 1,421,006-1,514,782 | 0 | 3R:29A | nd | 3R:29B |
|  | 2 | 11_H04 | BU038907 | AGAP007827 | 1,483,634-1,486,754 | 0 | 3R:29A | 2L:27D | nd |
| 5 |  | AsUbi | AJ415521 | AGAP007927  AGAP001971  AGAP008001 | 2,920,166-2,921,050  12,998,558-12,999,247  3,991,227-3,993,208 | 1e-100  1e-69  4e-66 | 3R:29C  2R:9C  3R:29D | nd | 3R:29AB |
| 6 |  | 109G18 | BH368579 |  | 3,963,987-4,060,834 | 0 | 3R:29CD | nd | 3R:29D |
|  | 3 | 01_C07 | BU038870 | AGAP008001 | 3,991,227-3,993,208 | 6e-84  1e-29 | 3R:29CD  2R:29D | 2L:27C | nd |
| 7 | 4 | 06_F07 | BU038888 | AGAP008053 | 4,869,662-4,871,563 | 1e-179 | 3R:29D | 2L:27B | 3R:29D |
| 8 | 5 | 16_C12 | BU038929 | AGAP008054 | 4,880,891-4,881,271 | 1e-162 | 3R:29D | 2L:27B | 3R:29D |
| 9 |  | 178A3 | BH398965 |  | 5,630,291-5,756,885 | 0 | 3R:29D | nd | 3R:29E*; X:6A(het); 2L:20AB(het) |
| 10 |  | 24K22 | AL152579 |  | 6,541,606-6,644,135 | 0 | 3R:30A | nd | 3R:30A |
|  | 6 | CYP6Z3 | AY193727 | AGAP008217 | 6,971,669-6,973,217 | 0 | 3R:30A | 2L:26D | nd |
|  | 7 | CYP6Z1 | AF487535 | AGAP008219 | 6,976,539-6,978,081 | 0 | 3R:30A | 2L:26D | nd |
| 11 |  | 23K05 | AL610371 |  | 7,477,602-7,608,857 | 0 | 3R:30AB | nd | 3R:30A |
|  | 8 | 21_D06 | BU038954 | AGAP008233 | 7,543,137-7,543,741 | 2e-25 | 3R:30AB | 2L:26D | nd |
|  | 9 | AFUB10 | AY029717 | AGAP008241 | 7,587,587-7,590,544 | 2e-32 | 3R:30B | 2L:26CD | nd |
| 12 |  | 145J17 | BH373436 |  | 8,596,001-8,683,047 | 0 | 3R:30B | nd | 3R:30A |
|  | 10 | 06_E04 | BU038886 | AGAP008294 | 8,630,719-8,631,546 | 2e-22 | 3R:30B | 2R, 18C 2L, 26C* | nd |
| 13 |  | 13J12 | AL147066 |  | 8,716,522-8,842,512 | 0 | 3R:30B | nd | 3R:30B |
|  | 11 | Fun P | AY116020 | AGAP008304 | 8,793,109-8,802,070 | 1e-40 | 3R:30B | 2L:26C | nd |
| 14 |  | 03N21 | AL141483 |  | 10,019,888-10,142,236 | 0 | 3R:30C | nd | 3R:30B |
|  | 12 | 13_H04 | BU038920 | AGAP008369 | 10,088,721-10,093,543 | 4e-64 | 3R:30C | 2L:26A | nd |
| 15 |  | AST021C18 | Z. Tu lab |  | 10,123,759-10,129,187 | 4e-78 | 3R:30C | nd | 3R:30C |
| 16 | 13 | AF264E05 | F.H. Collins lab |  | 10,774,634-10,774,684 | 9e-05 (35 bp) | 3R:30D | 2L:24B | 3R:31C |
| 17 |  | AsMad | AY578813 | AGAP008551 | 12,545,417-12,547,013. | 0 | 3R:30E | nd | 3R:32A |
| 18 | 14 | 61_F02 | BU039004 | AGAP008647 | 13,939,170-13,940,623 | 4e-55 | 3R:31A | 2L:22B | 3R:32C |
| 19 |  | 10F04 | AL145343 |  | 16,038,336-16,118,290 | 0 | 3R:31B | nd | 3R: 32C |
|  | 15 | 13_C02 | BU038917 | AGAP008725 | 16,069,773-16,071,472 | 8e-75 | 3R:31B | 2L:22D | nd |
| 20 |  | 211H04 | EX227579 | AGAP008727 | 16,158,263-16,159,216 | 1E-96 | 3R:31BC | nd | 3R:32C |
| 21 |  | 29A01 | AL611251 |  | 17,147,606-17,221,188 | 0 | 3R:31C | nd | 3R:33A |
|  | 16 | 23_B09 | BU038962 | AGAP008762 | 17,144,470-17,150,005 | 1e-57 | 3R:31C | 2L:23A | nd |
|  | 17 | AFUB2 | AY029709 |  | 17,154,074-17,154,296 | 5e-39 | 3R:31C | 2L:23A | nd |
| 22 |  | 31B02 | AL146601 |  | 19,607,644-19,703,771 | 0 | 3R:32A | nd | 3R:34B |
| 23 |  | 211E11 | EX227555 | AGAP008923 | 21,042,156-21,046,135 | 3e-14 | 3R:32C | nd | 3R:34B, 3R:29B* |
| 24 |  | 25M15 | AL153203 |  | 25,546,570-25,694,004 | 0 | 3R:33B | nd | 3R:36A |
|  | 18 | 98_F05 | BU039018 | AGAP009096 | 25,578,233-25,580,290 | 4e-61 | 3R:33B | 2L:24D | nd |
| 25 | 19 | AF264H03 | F.H. Collins lab |  | 25,983,063-25,983,530 | 2.0e-7 | 3R:33B | 2L:24C | 3R:36A |
|  | 20 | AFND18 | AF171048 |  | 26,560,353-26,560,412 | 7e-12 | 3R:33C | 2L:24C | nd |
|  | 21 | FUN L | AY116016 |  | 26,816,555-26,816,623 | 8.0e-18 | 3R:33C | 2L:24C | nd |
| 26 |  | 02J17 | AL140752 |  | 27,508,676-27,597,800 | 0 | 3R:33C |  | 3R:36A |
| 27 |  | AsSerpin6 | M. Jacobs-Lorena lab | AGAP009212 | 28,811,997-28,818,217 | 1e-128 | 3R:33C | nd | 3R:31C |
| 28 |  | 152P1 | BH375235 |  | 30,494,707-30,573,515 | 0 | 3R:33D | nd | 3R:30C |
| 29 |  | 135D12 | BH371101 |  | 31,230,315-31,384,115 | 0 | 3R:33D | nd | 3R:30C |
|  | 22 | 25_H10 | BU038975 | AGAP009324 | 31,268,756-31,270,480 | 7e-36 | 3R:33D | 2L:28C | nd |
| 30 |  | 14E16 | AL147453 |  | 31,471,236-31,630,052 |  | 3R:33D | nd | 3R:31A |
| 31 |  | AST018E12 | Z. Tu lab |  | 32,213,137-32,216,768 | 7e-12 | 3R:34A | nd | 3R:31A |
| 32 |  | 212B05 | EX227597 | AGAP009441 | 33,496,836-33,501,776 | 5e-66 | 3R:34A | nd | 3R:31A |
| 33 |  | 12A10 | AL146243 |  | 33,562,651-33,640,581 | 0 | 3R:34B | nd | 3R:31A |
| 34 | 23 | AF12D10 | F.H. Collins lab |  | 34,898,584-34,898,654 | 3.0e-14 | 3R:34B | 2L:28B | 3R:31A |
| 34 |  | 211H06 | EX227580 | AGAP009508 | 34,969,675-34,970,901 | 1e-113 | 3R:34B | nd | 3R:31A |
| 36 |  | 211C04 | EX227533 | AGAP009515 | 35,054,613-35,076,625 | 4e-33 | 3R:34BC | nd | 3R:31A |
| 37 |  | AST026I17 | Z. Tu lab |  | 35,131,422-35,136,776 | 2e-18 | 3R:34C | nd | 3R:31B |
|  | 24 | 66_E10 | BU039010 | AGAP009537 | 35,605,568-35,607,573 | 1e-130 | 3R:34C | 2L:28A | nd |
| 38 |  | 23J24 | AL152013 |  | 36,477,003-36,591,879 | 0 | 3R:34C | nd | 3R:31B |
|  | 25 | AFND23 | AY291367 |  | 36,927,785-36,927,839 | 3e-10 | 3R:34D | 2L:24A | nd |
| 39 |  | 211E10 | EX227554 | AGAP009610 | 37,001,849-37,003,632 | 7e-22 | 3R:34D | nd | 3R:32C |
| 40 |  | AST029K10 | Z. Tu lab |  | 37,408,194-37,420,606 | 2e-12 | 3R:34D | nd | 3R:33B |
| 41 |  | 23G11 | AL151883 |  | 38,599,533-38,718,166 | 0 | 3R:35B | nd | 3R:33C |
| 42 |  | 05C06 | AL607943 |  | 39,502,006-39,614,515 | 0 | 3R:35B | nd | 3R:31C |
| 43 |  | 163H10 | BH385794 |  | 40,492,051-40,582,084 | 0 | 3R:35B | nd | 3R:37B |
| 44 |  | 31B09 | AL156246 |  | 41,547,299-41,646,263 | 0 | 3R:35B | nd | 3R:35A*,  30B; 2L:25B |
| 45 |  | 30P20 | AL156192 |  | 42,487,765-42,579,439 | 0 | 3R:35C | nd | 3R:37B*, X:6A(het) |
| 46 |  | 125G23 | BH389922 |  | 43,532,477-43,646,278 | 0 | 3R:35C | nd | 3R:35B |
| 47 |  | 211B10 | EX227528 | AGAP009839 | 44,086,477-44,088,125 | 5e-24 | 3R:35D | nd | 3R:35B |
| 48 |  | 211F01 | EX227557 | AGAP009944 | 45,804,678-45,806,458 | 1e-39 | 3R:36AB | nd | 3R:36A |
| 49 |  | 129I2 | BH372692 |  | 46,515,634-46,627,757 | 0 | 3R:36B | nd | 3R: |
| 50 |  | 11I19 | AL146017 |  | 48,498,702-48,595,676 | 0 | 3R:36D | nd | 37B |
| 51 | 26 | 61_G06 | BU039005 | AGAP010142 | 49,370,093-49,372,300 | 2e-59 | 3R:37A | 2L:20D | 3R:37B |
|  | 27 | 11_C01 | BU038901 | AGAP010207 | 50,715,566-50,720,049 | 3e-45 | 3R:37C | 2L:20C | nd |
| 52 |  | 211A05 | EX227515 | AGAP010216 | 50,847,429-50,848,247 | 7e-43 | 3R:37C | nd | 3R:37C |
|  | 28 | 19_D07 | BU038946 | AGAP010216 | 50,847,429-50,848,247 | 3e-89 | 3R:37C | 2L:20C*;  3L:44D,46A | 3R:37C |
| 53 |  | 627112 | BM636978 | AGAP010252 | 51,662,576-51,663,611 | 0 | 3R:37D | nd | 3R:37D |
|  | 29 | 66_A04 | BU039008 | AGAP010252 | 51,662,576-51,663,611 | 6e-33 | 3R:37D | 2L:20B | nd |
| Markers on 3L | | | | | | | | | |
| Markers in *An. stephensi* | Markers in *An. funestus* | Clone  name | Accession | VectorBase Gene ID | Genomic Location in  *An. gambiae* | e-value | Chromosomal location | | |
|  |  |  |  |  |  |  | *An. gambiae* | *An. funestus* | *An. stephensi* |
|  | 1 | 15_B11 | BU038923 | AGAP10387 | 2,444,111-2,447,808 | 4e-95 | 3L:38B | 3L:38C | nd |
| 1 |  | 212D01 |  | AGAP010364 | 2,246,808-2,247,865 | 9e-09 | 3L:38B | nd | 2L:20C |
| 2 |  | 148K2 | BH396659 |  | 2,709,606-2,840,913 | 0 | 3L:38B | nd | 2L:20C |
|  | 2 | 01_F07 | BU038872 | AGAP010404 | 2,779,188-2,784,335 | 7e-69 | 3L:38B | 3L:38C | nd |
| 3 | 3 | 07_G04 | BU038892 | AGAP010445 | 3,752,432-3,757,036 | 3e-73 | 3L:38C | 3L:39A | 2L:20C |
|  | 4 | 25_E01 | BU038971 | AGAP10469 | 4,015,796-4,031,373 | 1e-42 | 3L:38C | 3L:39A | nd |
| 4 |  | 139K20 | BH402428 |  | 4,607,484-4,702,647 | 0 | 3L:38C | nd | 2L:20A* |
| 5 |  | 151M24 | BH399147 |  | 4,982,245-5,081,675 | 0 | 3L:38C | nd | 2L:22A |
|  | 5 | 27_B04 | BU038981 | AGAP010500 | 5,056,173-5,058,317 | 3e-41 | 3L:38C | 3L:39A*,46A | nd |
| 6 |  | 105O20 | BH379197 |  | 5,545,686-5,611,702 | 0 | 3L:39A | nd | 2L:22B |
| 7 |  | 139M22 | BH387916 |  | 6,371,454-6,495,502 | 0 | 3L:39A | nd | 2L:22B |
|  | 6 | 29_D12 | BU038986 | AGAP010565 | 6,421,493-6,424,472 | 1e-102 | 3L:39A | 3L:39B | nd |
| 8 | 7 | 03_G10 | BU038875 | AGAP010657 | 8,102,799-8,116,056 | 9e-61 | 3L:39B | 3L:41A | 2L:22C |
|  |  | 36_D10 | BU038999 | AGAP010657 | 8,102,799-8,116,056 | 1e-152 | 3L:39B | 3L:41A | 2L:22C |
| 9 |  | 126G21 | BH375705 |  | 8,852,435-8,989,815 | 0 | 3L:39C | nd | 2L:21A |
|  | 8 | 12_B09 | BU038909 | AGAP010716 | 8,934,066-8,937,311 | 1e-60 | 3L:39C | 3L:41A | nd |
| 10 |  | 180K21 | BH367855 |  | 9,407,395-9,639,192 | 0 | 3L:39C | nd | 2L:25B*; 3R:36C |
|  | 9 | 15_F08 | BU038924 | AGAP010792 | 10,412,154-10,413,607 | 1e-171 | 3L:40A | 3L:43A | nd |
| 11 |  | AST012A11 | Z. Tu lab |  | 11,244,143-11,260,272 | 8e-19 | 3L:40B | nd | 2L:28C |
| 12 | 10 | 12_F01 | BU038911 |  | 11,726,542-11,727,596 | 1e-91 | 3L:40B | 3L:43B | 2L:28C |
| 13 |  | 105F8 | BH392724 |  | 12,527,507-12,636,192 |  | 3L:40B | nd | 3R:37B; 2L:25B* |
| 14 |  | 02G07 | AL140620 |  | 13,725,402-13,907,560 | 0 | 3L:40C | nd | 2L:25B |
| 15 |  | 31H07 | AL156465 |  | 14,419,173-14,419,723 | 0 | 3L:41A | nd | 2L:21A,  26A* |
| 16 |  | 08F18 | AL144178 |  | 14,578,788-14,693,933 | 0 | 3L:41A | nd | 2L:27C |
| 17 |  | 25B13 | AL152825 |  | 16,548,366-16,667,678 | 0 | 3L:41B | nd | 2L:27C |
| 18 |  | 130M5 | BH384886 |  | 17,484,256-17,609,908 | 0 | 3L:41C | nd | 2L:27B |
| 19 |  | 650820 | BM650357 | AGAP011160 | 18,112,740-18,113,828 | 0 | 3L:41C | nd | 2L:27C |
|  | 11 | 36_A01 | BU038992 | AGAP011160 | 18,112,740-18,113,828 | 1e-162 | 3L:41C | 3L:42B | nd |
| 20 |  | AST041D2 | Z. Tu lab |  | 18,440,434-18,526,417 | 5e-29 | 3L:41D | nd | 2L:27A |
| 21 |  | 27P23 | AL154485 |  | 20,554,497-20,635,434 | 0 | 3L:42B | nd | 2L:25B |
|  | 12 | 21_F09 | BU038957 | AGAP011291 | 20,578,594-20,579,662 | 1e-79 | 3L:42B | 3L:44D | nd |
| 22 |  | 211A03 | EX227514 | AGAP011298 | 20,688,570-20,689,982 | 7e-36 | 3L:42B | nd | 2L:25B |
|  | 13 | 29_F07 | BU038989 | AGAP011402 | 23,869,077-23,869,479 | 3e-74 | 3L:42C | 3L:44A*,  2R: 17B | nd |
| 23 |  | 155I2 | BH374558 |  | 23,877,430-23,986,176 |  | 3L:43A | nd | 2L:24B,  24A* |
| 24 |  | 145G13 | BH370252 |  | 24,356,682-24,417,969 |  | 3L:43A | nd | 2L:24A*; 3R:37B |
| 25 |  | 04F19 | AL141759 |  | 26,250,362-26,366,741 | 0 | 3L:43B | nd | 2L:24B |
|  | 14 | 04_D01 | BU038876 | AGAP011514 | 26,294,061-26,295,679 | 1e-36 | 3L:43B-gene | 3L:46D | 2L:24B |
| 26 |  | 124K17 | BH372801 |  | 27,566,602-27,673,312 |  | 3L:43C | nd | 2L:24B |
| 27 |  | 12I09 | AL146524 |  | 28,283,793-28,363,998 | 0 | 3L:43C | nd | 2L:24C |
|  | 15 | 27_A08 | BU038980 | AGAP011581 | 28,373,084-28,373,665 | 2e-87 | 3L:43C | 3L:46B*; X:4B,3 | nd |
| 28 | 16 | AF13G04 | F.H. Collins lab |  | 30,431,704-30,431,754 | 1e-10 | 3L:43D | 3L:45C | 2L:23A |
| 29 |  | 211D02 | EX227540 | AGAP011644 | 30,654,707-30,670,454 | 5.3e-66 | 3L:43D | nd | 2L:23B |
| 30 | 17 | AF263D12 | F.H. Collins lab |  | 32,057,098-32,057,136 | 1.1e-6 | 3L:44A | 3L:45B | 2L:23B |
| 31 |  | 11G16 | AL145956 |  | 32,599,213-32,701,072 | 0 | 3L:44B | nd | 2L:23C |
| 32 |  | 669234 | BM655755 | AGAP011788 | 33,271,412-33,273,378 | 6e-44 | 3L:44B | 3L:40A | 2L:21B-20A*  3R:37D |
|  | 18 | 25_H01 | BU038974 |  |  |  |  |  |  |
| 33 | 19 | 04_E02 | BU038879 | AGAP011828 | 33,872,056-33,877,105 | 1e-176 | 3L:44C | 3L:40B | 2L:21B |
| 34 |  | 29L12 | AL155478 |  | 33,874,267-33,982,382 | 0 | 3L:44C | nd | 2L:21B |
|  | 20 | 21_G01 | BU038959 | AGAP011839 | 33,925,153-33,927,790 | 5e-42 | 3L:44C | 3L:40B | nd |
| 35 |  | 131K7 | BH378526 |  | 34,547,460-34,646,125 | 0 | 3L:44D | nd | 2L:21B |
| 36 |  | AsGbb | AY578815 | AGAP011934 | 35,258,637-35,260,057 | 2e-88 | 3L:44D | nd | 2L: 21B |
| 37 |  | 01K17 | AL140205 |  | 35,505,997-35,582,996 | 0 | 3L:44D | nd | 2L:21A |
| 38 |  | 132D12 | BH373552 |  | 37,599,013-37,651,873 | 0 | 3L:45C | nd | 2L:26B |
|  | 21 | 20_G09 | BU038953 | AGAP012096 | 37,631,554-37,633,381 | 1e-101 | 3L:45C | 3L:41B*, 3R:29C | nd |
|  | 22 | 19_H06 | BU038949 | AGAP012131 | 37,960,351-37,965,533 | 2e-42 | 3L:45C | 3L:40C | nd |
| 39 |  | 104F13 | BH377291 |  | 37,971,196-38,080,288 | 0 | 3L:45C | nd | 2L:26C |
|  | 23 | 05_A10 | BU038880 | AGAP012334 | 40,732,639-40,738,864 | 7e-47 | 3L:46C | 3L:44A | nd |
| 40 |  | 28J05 | AL154795 |  | 40,982,673-41,158,235 | 0 | 3L:46C | nd | 2L:28A |
| 41 |  | 28G12 | AL154707 |  | 41,538,346-41,662,654 | 0 | 4L:46D | nd | 2L:28A |
| 42 |  | 126M2 | BH386745 |  | 41,792,338-41,892,789 | 0 | 3L:46D | nd | 2L:28A |
|  | 24 | 36_C05 | BU038998 | AGAP012418 | 41,834,603-41,835,368 | 1e-44 | 3L:46D | 3L:46B | nd |

*) Asterisks indicate primary BLAST hits and strongest hybridization signals.
