# Supplementary material for: Genome Landscape and Evolutionary Plasticity of Chromosomes in Malaria Mosquitoes
Source: PLoS One. 2010 May 12;5(5):e10592. doi: 10.1371/journal.pone.0010592 (PMC2868863; doi:10.1371/journal.pone.0010592)
Supplement: Table S2 — Measures of uniformity of marker distribution for An. gambiae, An. stephensi, and An. funestus. (0.06 MB DOC) [file pone.0010592.s006.doc]

**Table S2. Measures of uniformity of marker distribution for *An. gambiae*, *An. stephensi*, and *An. funestus*.**

| *An. gambiae* | | | | |
| --- | --- | --- | --- | --- |
| Arm | X^2^ | Asymptotic p | Bootstrap p | N |
| X | 6.80 | 0.138 | 0.147 | 5 |
| 2R | 8.06 | 0.714 | 0.708 | 12 |
| 2L | 2.97 | 0.933 | 0.936 | 9 |
| 3R | 5.10 | 0.826 | 0.832 | 10 |
| 3L | 4.00 | 0.780 | 0.790 | 8 |
| *An. stephensi* | | | | |
| Arm | X^2^ | Asymptotic p | Bootstrap p | N |
| X | 2.60 | 0.660 | 0.637 | 5 |
| 2R | 4.01 | 0.780 | 0.793 | 12 |
| 2L | 7.55 | 0.765 | 0.753 | 9 |
| 3R | 11.34 | 0.253 | 0.256 | 10 |
| 3L | 6.00 | 0.792 | 0.792 | 8 |
| *An. funestus* | | | | |
| Arm | X^2^ | Asymptotic p | Bootstrap p | N |
| X | 0.692 | 0.875 | 0.928 | 5 |
| 2R | 1.353 | 0.929 | 0.921 | 7 |
| 2L | 4.276 | 0.375 | 0.375 | 6 |
| 3R | 5.407 | 0.248 | 0.241 | 6 |
| 3L | 2.5 | 0.448 | 0.475 | 5 |
