# Supplementary material for: Genome Landscape and Evolutionary Plasticity of Chromosomes in Malaria Mosquitoes
Source: PLoS One. 2010 May 12;5(5):e10592. doi: 10.1371/journal.pone.0010592 (PMC2868863; doi:10.1371/journal.pone.0010592)
Supplement: Table S3 — Inversion fixation rates between An. funestus and An. gambaie calculated by GRIMM from the gene order. (0.05 MB DOC) [file pone.0010592.s007.doc]

**Table S3. Inversion fixation rates between *An. funestus* and *An. gambaie* calculated by GRIMM from the gene order.**

| Chromosome arm | The number of inversions, *n* | The length of chromosomal arm, *G* (Mb) | The number of inversions per  1 Mb | The number of breaks per  1 Mb |
| --- | --- | --- | --- | --- |
| X | 8 | 24.393 | 0.328 | 0.656 |
| 2R | 16 | 61.545 | 0.260 | 0.520 |
| 2L | 10 | 49.364 | 0.203 | 0.405 |
| 3R | 6 | 53.201 | 0.113 | 0.226 |
| 3L | 7 | 41.963 | 0.167 | 0.334 |
