# Supplementary material for: Genome Landscape and Evolutionary Plasticity of Chromosomes in Malaria Mosquitoes
Source: PLoS One. 2010 May 12;5(5):e10592. doi: 10.1371/journal.pone.0010592 (PMC2868863; doi:10.1371/journal.pone.0010592)
Supplement: Table S4 — Posterior estimates for the mean length of each conserved segment (L, Mb) for each of the chromosome arms and the whole genome. (0.05 MB DOC) [file pone.0010592.s008.doc]

**Table S4. Posterior estimates for the mean length of each conserved segment (L,** Mb) for each of the chromosome arms and the whole genome.

| Arm | E[L|D] | SE[L|D] | 95% credible interval | MAP |
| --- | --- | --- | --- | --- |
| X | 0.892734 | 0.493514 | (0.365225, 2.175180) | 0.600050 |
| 2R | 1.436172 | 0.302855 | (0.964647, 2.150852) | 1.315324 |
| 2L | 2.041102 | 0.665096 | (1.156230, 3.748442) | 1.712251 |
| 3R | 4.223825 | 1.185410 | (2.379823, 6.979740) | 3.755731 |
| 3L | 2.931428 | 0.951138 | (1.595421, 5.291594) | 2.412339 |
| All | 2.003 | 0.253 | (1.570, 2.562) | 1.943 |
