# Supplementary material for: Genome Landscape and Evolutionary Plasticity of Chromosomes in Malaria Mosquitoes
Source: PLoS One. 2010 May 12;5(5):e10592. doi: 10.1371/journal.pone.0010592 (PMC2868863; doi:10.1371/journal.pone.0010592)
Supplement: Table S5 — Density and coverage of molecular elements in chromosomal arms of An. gambiae. (0.08 MB DOC) [file pone.0010592.s009.doc]

**Table S5. Density and coverage of molecular elements in chromosomal arms of *An. gambiae*.**

| DNA transposons | | | | | |
| --- | --- | --- | --- | --- | --- |
|  | X | 2R | 2L | 3R | 3L |
| Mean densities | 156.630 | 71.644 | 119.757 | 76.496 | 113.127 |
| Median densities | 162.500 | 40.200 | 77.606 | 64.300 | 91.200 |
| Mean ranks | 3.748 | 2.251 | 3.108 | 2.729 | 3.164 |
| RNA transposons | | | | | |
|  | X | 2R | 2L | 3R | 3L |
| Mean densities | 188.976 | 116.565 | 156.383 | 118.213 | 141.695 |
| Median densities | 178.696 | 69.800 | 132.100 | 96.298 | 128.947 |
| Mean ranks | 3.470 | 2.501 | 3.158 | 2.766 | 3.105 |
| Segmental duplications | | | | | |
|  | X | 2R | 2L | 3R | 3L |
| Mean densities | 12.418 | 15.233 | 17.404 | 11.580 | 15.546 |
| Median densities | 12.472 | 16.180 | 12.563 | 8.000 | 13.900 |
| Mean ranks | 2.874 | 3.145 | 2.961 | 2.783 | 3.237 |
| Microsatellites | | | | | |
|  | X | 2R | 2L | 3R | 3L |
| Mean coverage | 0.989 | 0.437 | 0.445 | 0.391 | 0.400 |
| Median coverage | 0.985 | 0.420 | 0.435 | 0.390 | 0.385 |
| Mean ranks | 4.966 | 2.564 | 2.748 | 2.362 | 2.360 |
| Minisatellites | | | | | |
|  | X | 2R | 2L | 3R | 3L |
| Mean coverage | 1.634 | 0.923 | 1.206 | 1.114 | 1.109 |
| Median coverage | 1.670 | 0.660 | 1.020 | 0.860 | 0.865 |
| Mean ranks | 4.112 | 2.359 | 2.886 | 2.759 | 2.883 |
| Satellites | | | | | |
|  | X | 2R | 2L | 3R | 3L |
| Mean coverage | 0.544 | 0.298 | 0.421 | 0.269 | 0.214 |
| Median coverage | 0.565 | 0.230 | 0.255 | 0.180 | 0.140 |
| Mean ranks | 4.315 | 2.627 | 3.063 | 2.579 | 2.416 |
| MARs | | | | | |
|  | X | 2R | 2L | 3R | 3L |
| Mean coverage | 4.015 | 3.533 | 4.923 | 4.486 | 5.401 |
| Median coverage | 4.395 | 2.900 | 5.385 | 5.000 | 6.000 |
| Mean ranks | 2.701 | 2.344 | 3.326 | 3.054 | 3.575 |
| Genes | | | | | |
|  | X | 2R | 2L | 3R | 3L |
| Mean densities | 31.921 | 54.056 | 50.125 | 39.868 | 44.052 |
| Median densities | 28.685 | 59.400 | 57.600 | 43.644 | 48.445 |
| Mean ranks | 2.218 | 3.806 | 3.479 | 2.576 | 2.921 |
